# iPSC-derived mesenchymal cells that support alveolar organoid development

**DOI:** 10.1016/j.crmeth.2022.100314

**Published:** 2022-09-19

**Authors:** Koji Tamai, Kouji Sakai, Haruka Yamaki, Keita Moriguchi, Koichi Igura, Shotaro Maehana, Takahiro Suezawa, Kazuaki Takehara, Masatoshi Hagiwara, Toyohiro Hirai, Shimpei Gotoh

**Affiliations:** 1 Department of Respiratory Medicine, Graduate School of Medicine, Kyoto University, Kyoto, Japan; 2 Department of Veterinary Science, National Institute of Infectious Diseases, Tokyo, Japan; 3 Department of Virology 3, National Institute of Infectious Diseases, Tokyo, Japan; 4 Department of Drug Discovery for Lung Diseases, Graduate School of Medicine, Kyoto University, Kyoto, Japan; 5 Department of Environmental Microbiology, Graduate School of Medical Sciences, Kitasato University, Kanagawa, Japan; 6 Department of Microbiology, School of Allied Health Sciences, Kitasato University, Kanagawa, Japan; 7 Regenerative Medicine and Cell Design Research Facility, Kanagawa, Japan; 8 Laboratory of Animal Health, Department of Veterinary Medicine, Faculty of Agriculture, Tokyo University of Agriculture and Technology, Tokyo, Japan; 9 Laboratory of Animal Health, Cooperative Division of Veterinary Science, Graduate School of Agriculture, Tokyo University of Agriculture and Technology, Tokyo, Japan; 10 Department of Anatomy and Developmental Biology, Graduate School of Medicine, Kyoto University, Kyoto, Japan; 11 Center for iPS Cell Research and Application (CiRA), Kyoto University, Kyoto, Japan

**Keywords:** mesenchymal cell, pluripotent stem cell, lung, organoid, influenza virus, SARS-CoV-2

## Abstract

Mesenchymal cells are necessary for organ development. In the lung, distal tip fibroblasts contribute to alveolar and airway epithelial cell differentiation and homeostasis. Here, we report a method for generating human induced pluripotent stem cell (iPSC)-derived mesenchymal cells (iMESs) that can induce human iPSC-derived alveolar and airway epithelial lineages in organoids via epithelial-mesenchymal interaction, without the use of allogenic fetal lung fibroblasts. Through a transcriptome comparison of dermal and lung fibroblasts with their corresponding reprogrammed iPSC-derived iMESs, we found that iMESs had features of lung mesenchyme with the potential to induce alveolar type 2 (AT2) cells. Particularly, RSPO2 and RSPO3 expressed in iMESs directly contributed to AT2 cell induction during organoid formation. We demonstrated that the total iPSC-derived alveolar organoids were useful for characterizing responses to the influenza A (H1N1) virus and severe acute respiratory syndrome coronavirus 2 (SARS-CoV-2) infection, demonstrating their utility for disease modeling.

## Highlights

• We report a method for generating iPSC-derived mesenchymal cells (iMESs)
• iMESs induce development of alveolar organoids without the use of allogenic fetal cells
• iMES-alveolar organoids enable studies of the niche environment and pulmonary diseases

## Motivation

Organoid technology is a powerful tool for bioscience research. Previously, human fetal lung fibroblasts (HFLFs) have been used to promote the development of alveolar organoids, but HFLFs have the limitation of being allogenic in organoid systems, and therefore they cannot reproduce an individual’s biological environment. To address this limitation, we generated induced pluripotent stem cell-derived mesenchymal cells (iMESs) that have the ability to develop alveolar organoids with the aim of modeling niche environments and diseases.

## Graphical abstract

**Figure:**
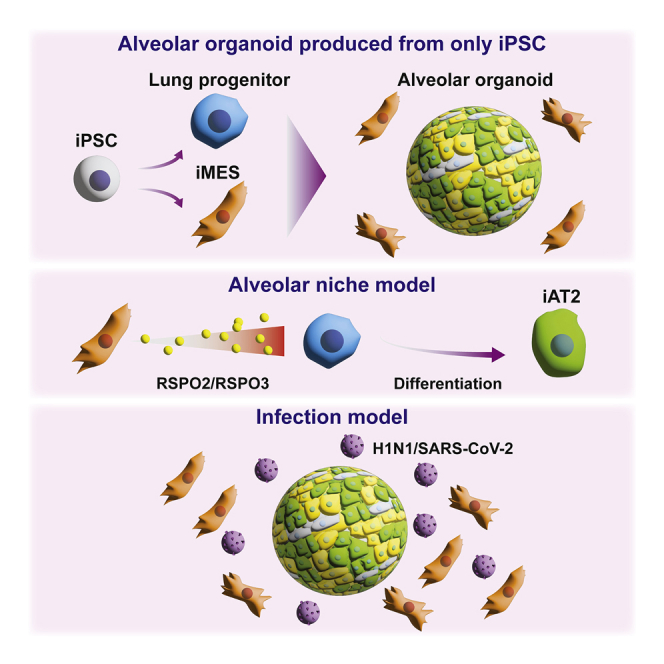

## Teaser

Tamai et al. develop a method for generating iPSC-derived mesenchymal cells (iMESs) capable of developing alveolar organoids (iMES-AOs). iMESs can induce not only alveolar epithelial type II but also type I cells. iMES-AOs provide a platform for modeling lung development and diseases including respiratory viral infection.

## Introduction

Mesenchymal cells provide extracellular matrix proteins and various secreted proteins suited to cell-type-specific tissue microenvironments, and their interaction with epithelial cells is essential for normal organ development, homeostasis, and regeneration. Previous studies have described human pluripotent stem cell (PSC)-derived alveolar epithelial cells in both a fibroblast-dependent (Gotoh et al., 2014; Yamamoto et al., 2017) and a fibroblast-free procedure (Jacob et al., 2017; Yamamoto et al., 2017). Challenges to simultaneous differentiation of lung epithelial and mesenchymal cells have been reported (Chen et al., 2017; Dye et al., 2015; Miller et al., 2019), but the process of deriving mesenchymal cells from PSCs remains unknown. Given the potential application of the PSC-derived alveolar organoid (AO) for research in human developmental processes and disease modeling, AOs should include mesenchymal cells. Primary human fetal lung fibroblasts (HFLFs) have been used, but it is often difficult to recapitulate the exact biological environment found in the lung. Although others have reported human PSC-derived lung mesenchymal cells that recapitulated the developmental course of mouse early fetal foregut organogenesis (Han et al., 2020; Kishimoto et al., 2020), AO formation using PSC-derived lung mesenchymal cells has not been accomplished. Hence, it is desirable to generate mesenchymal cells that can support organ development and facilitate further research in embryogenic organogenesis.

In this study, we report a method for generating human induced PSC (iPSC)-derived mesenchymal cells (iMESs) that are able to form AOs (iMES-AOs). We also explored niche factors to induce iPSC-derived alveolar type 2 (iAT2) cells from progenitor cells using transcriptomic analysis of paired isogenic iMESs and the mesenchymal cells of primary fibroblasts. Moreover, we used iMES-AOs in two pandemic respiratory infection models, the influenza A (H1N1) virus and severe acute respiratory syndrome coronavirus 2 (SARS-CoV-2).

## Results

### Generation of mesenchymal cells that induce iAT2 cells

**Figure 1:**
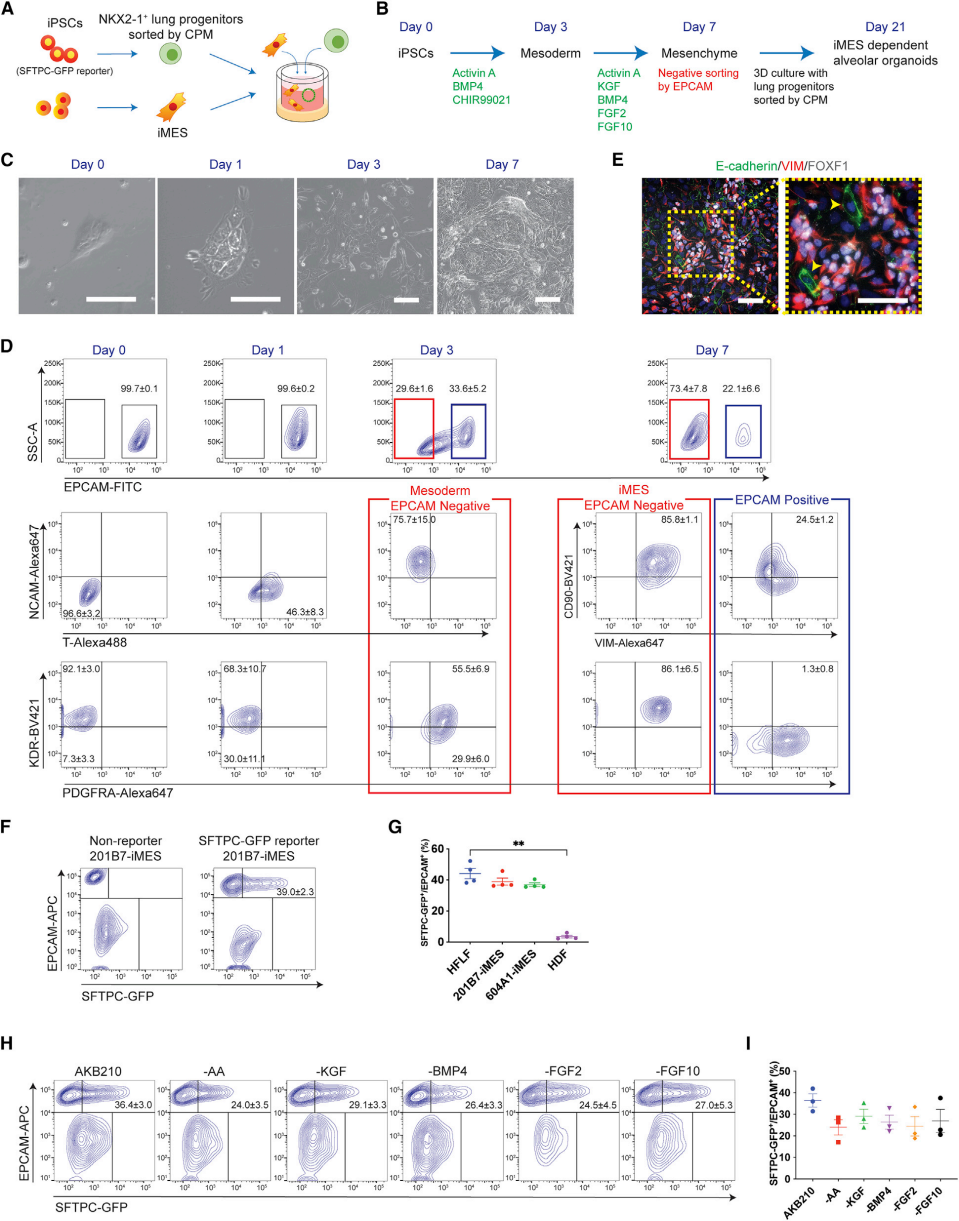
Targeted differentiation of iMESs capable of producing AOs (A) Strategy of producing AOs consisting of iPSC-derived lung progenitors and iMESs. (B) Schematic for stepwise induction of iMESs derived from iPSCs. (C) Phase-contrast images of cells during the differentiation process. Scale bars, 100 μm. (D) Flow cytometry data of representative markers during differentiation from 201B7 iPSCs. (E) Immunofluorescence staining on day 7. Scale bars, 100 μm. Arrowheads: E-cadherin^+^/FOXF1^−^ cells. (F and G) Flow cytometry data of the induction efficiency of SFTPC-GFP^+^/EPCAM^+^ cells and its quantification. iMESs were differentiated from 201B7 and 604A1 iPSCs. HFLFs and HDFs were used as positive and negative controls, respectively. The lung progenitors were differentiated from an SFTPC-GFP reporter iPSC line (B2-3). Negative control data were recorded using AOs consisting of lung progenitors and iMESs, both derived from the non-reporter 201B7. Data are presented as mean ± SEM. ∗∗p < 0.01 (Dunn’s post hoc test). (H and I) Effect on SFTPC-GFP induction by removing individual factors from the iMES differentiation medium between days 3 and 7. See also Figure S1.

We optimized a method to differentiate mesenchymal cells that were able to induce SFTPC^+^ AT2 lineage cells from their progenitor cells (Figures 1A and 1B). AOs were generated in a three-dimensional (3D) co-culture of SFTPC-GFP reporter iPSC (B2-3)-derived NKX2-1^+^ lung progenitors sorted by carboxypeptidase M (CPM) and iMESs derived from their parental 201B7 iPSC line (201B7-iMESs). First, we focused on mesoderm induction followed by differentiation of mesenchymal cells. On day 1, cell clusters started to lose their border sharpness in a medium containing activin A, BMP4, and CHIR99021, and they appeared as primitive streak-like cells expressing T-box T in the EPCAM^+^ cell population (Figures 1C and 1D). Cells became oblong in shape on day 3, and the EPCAM^−^ cell population became positive for the mesodermal markers NCAM, PDGFRα, and KDR (Evseenko et al., 2010; Sakurai et al., 2006). A new medium containing activin A, KGF, BMP4, FGF2, and FGF10 induced the expression of VIM, THY1, PDGFRα, and KDR on day 7. Because a minor EPCAM^+^ cell population showed insufficient mesenchymal marker expression (Figure 1D), we purified EPCAM^−^ cells and named them “iMESs.” Time course changes of each marker were validated using qRT-PCR, and gene expression levels were compared among iMESs, HFLFs, and human dermal fibroblasts (HDFs) (Figure S1A). iMESs also expressed FOXF1 and TBX4 as lung mesenchymal markers (Han et al., 2020; Horie et al., 2018) (Figures 1E and S1A).

3D co-culture of lung progenitors derived from B2-3 line and 201B7-iMESs produced spheroids that included SFTPC-GFP^+^ iAT2 cells (Figure S1B). Quantitative analysis using flow cytometry validated the SFTPC-GFP^+^/EPCAM^+^ iAT2 cell ratio in co-culture with HFLFs, 201B7-iMESs, and iMESs derived from another healthy donor-derived iPSC line (604A1) (Figures 1F and 1G). HDFs did not form spheroids and had fewer SFTPC-GFP^+^/EPCAM^+^ cells. We found that activin A, KGF, BMP4, FGF2, and FGF10 all affected iMESs and contributed to the induction of SFTPC-GFP^+^/EPCAM^+^ iAT2 cells. Each single factor was removed from the medium between days 3 and 7; AOs were produced with lung progenitors derived from B2-3 line and 201B7-iMESs, followed by quantification of the SFTPC-GFP^+^/EPCAM^+^ iAT2 cell ratio. iMESs treated with all factors induced the highest SFTPC-GFP^+^/EPCAM^+^ iAT2 cell ratio (Figures 1H and 1I). Immunofluorescence staining of iMES-AO samples showed that VIM^+^ iMESs spread throughout the spheroids. AT2 cell markers Pro-SPC, ABCA3, SFTPC-GFP, and mature-SPC were detected in the cuboidal cells. PDPN^+^/HT1-56^+^ thin-shaped cells were also observed, indicating the presence of iAT1 cells in the spheroids (Dobbs et al., 1999) (Figure S1C). AT1 and AT2 markers were detected in iMES-AOs using qRT-PCR (Figure S1D).

### Generation of iMESs from HFLF- and HDF-derived iPSCs to form AOs

**Figure 2:**
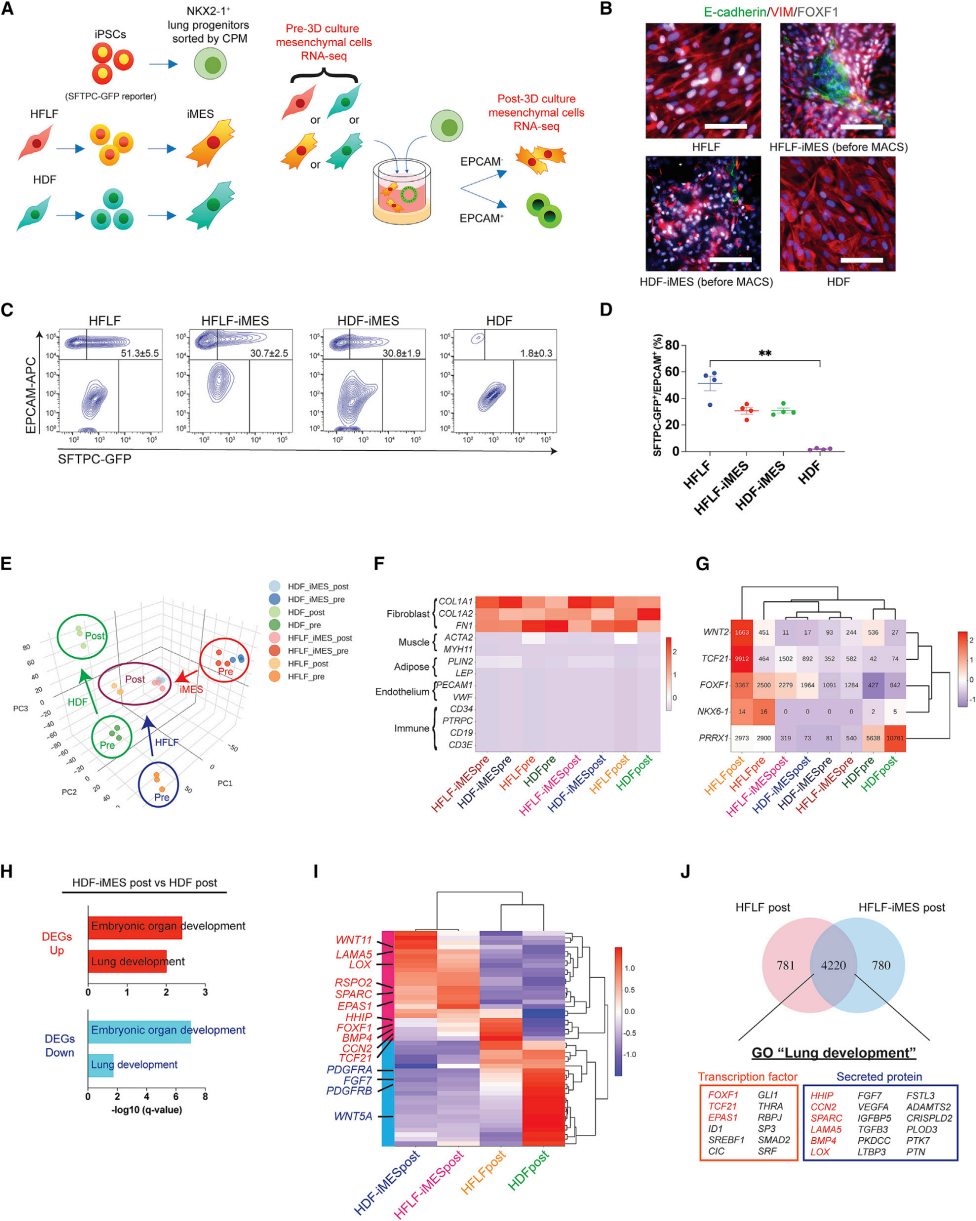
Generation of iPSCs from HFLFs and HDFs to compare the difference between iMESs and their original human primary fibroblasts (A) Strategy for pairwise isogenic comparison among HFLFs and HDFs (TIG120), and iMESs derived from iPSCs generated from each fibroblast. (B) Immunofluorescence staining of E-Cadherin, VIM, and FOXF1. Scale bars, 100 μm. (C and D) Flow cytometry data of the induction efficiency of SFTPC-GFP^+^/EPCAM^+^ cells and its quantification. iMESs were differentiated from HFLF-iPSCs (HFA) and HDF-iPSCs (GC23). The SFTPC-GFP reporter iPSC line (B2-3) was differentiated to lung progenitors. Data are presented as mean ± SEM. ∗∗p < 0.01 (Dunn’s post hoc test). (E) Principal-component analysis of RNA-seq transcriptomes of iMESs, HFLFs, and HDFs in each condition. (F) Heatmap of non-epithelial lineage marker genes. (G) Heatmap of selected respiratory mesenchymal markers. The numbers in cells are mean read counts of 3 replicates. (H) GO analysis of DEGs between HDF-iMESs versus HDFs in post-3D culture conditions. Representative GOs of biological processes in up-regulated (log_2_ fold change [FC] > 1, FDR < 0.05) and down-regulated DEGs (log_2_FC < −1, FDR < 0.05) are shown. (I) Heatmap of DEGs annotated to “lung development.” DEGs with a maximum average TPM of 3 replicates >20 between HDF-iMESs and HDFs in post-3D culture conditions were selected. Red and blue in the left side bar show up-regulated and down-regulated DEGs, respectively. (J) Common genes among the top 5,000 genes of HFLF-iMESs and HFLFs in post-3D culture conditions. Up-regulated transcription factors and secreted proteins annotated to “lung development” out of 4,220 genes in common are listed. Red colored genes indicate up-regulated genes in (I) as well. See also Figure S2.

To elucidate the role of iMESs in inducing iAT2 cells, we compared primary fibroblasts with iMESs. Because HDFs cannot generate AOs, we can eliminate unnecessary factors for AO development. We generated iPSCs from HFLFs and HDFs (HFLF-iPSCs and HDF-iPSCs, respectively) that presented expression of undifferentiated markers, normal karyotypes, and trilineage-differentiation potentials (Figures S2A and S2B). Both pre-and post-3D culture samples of HFLFs, HDFs, and their iMESs were recovered (Figure 2A). FOXF1 was highly expressed in HFLFs and each iMES but was weak in HDFs (Figure 2B). The ability to induce SFTPC-GFP^+^/EPCAM^+^ iAT2 cells was verified in both HFLF- and HDF-iMESs (Figures 2C and 2D).

### iMESs and HFLFs, but not HDFs, express genes associated with lung development

Principal-component analysis of RNA sequencing (RNA-seq) transcriptomes revealed well-separated clusters of each condition. Post-3D culture iMES and HFLF transcriptomes were both similar to one another and dissimilar to post-3D culture HDFs, inferred from the relative plot distance between populations (Figure 2E). Selected lineage gene markers for fibroblast, muscle, adipocyte, endothelial, and immune cells were depicted on a heatmap in columns scaled to transcripts per million (TPM). The expression levels of fibroblast markers were considerably high, indicating that iMESs share transcriptomic programs similar to fibroblasts (Figure 2F). Furthermore, we evaluated respiratory mesenchymal markers, including *WNT2*, *TCF21*, *FOXF1*, *NKX6-1*, and *PRRX1* (Goss et al., 2009; Han et al., 2020; Kishimoto et al., 2020; Park et al., 2019; Yeo et al., 2018) (Figure 2G). Post-3D culture HFLFs showed the highest expression of *WNT2*, followed by pre-3D culture HFLFs and HDFs, but HFLF-iMESs, HDF-iMESs, and post-3D HDFs expressed WNT2 at extremely low levels. Although expression levels of *TCF21* and *FOXF1* were also highest in post-3D culture HFLFs, they were also prominent in post-3D culture HFLF- and HDF-iMESs. *NKX6-1* was barely detected in any of the samples, and *PRRX1* was highest in HDFs, followed by HFLFs. Gene Ontology (GO) enrichment analysis of biological processes indicated that “embryonic organ development” and “lung development” were significantly enriched in both up- and down-regulated differentially expressed genes (DEGs) between HDF-iMESs and HDFs (Figure 2H). DEGs annotated to “lung development” were illustrated in a heatmap using four groups of mesenchymal transcriptomes (Figure 2I). Although *WNT5A*, *FGF7*, and *PDGFRA* are important factors in AT2 cells (Barkauskas et al., 2013; Nabhan et al., 2018; Zepp et al., 2017), they were up-regulated in HDFs. Secreted factors, including *RSPO2*, *WNT11*, *CCN2*, *SPARC*, *BMP4*, *HHIP*, *LAMA5*, and *LOX* were up-regulated in iMESs. Expression levels of transcription factors, including *FOXF1* and *TCF21*, were higher in HFLF- and HDF-iMESs and HFLFs than in HDFs, suggesting that iMESs share features of fetal lung fibroblasts. HFLF-iMESs and HFLFs shared expression of *EPAS1*, yet HDFs expressed *EPAS1* at higher levels than HFLFs, which indicates that *EPAS1* is not specific to the lung mesenchyme (Figure 2I). Next, we compared the top 5,000 genes of post-3D culture HFLF-iMESs and HFLFs in a Venn diagram (Figure 2J). There were 4,220 common genes, of which “lung development” was enriched (false discovery rate [FDR] q = 0.001). Genes annotated to “lung development” included *HHIP*, *CCN2*, *SPARC*, *BMP4*, *LAMA5*, and *LOX*, suggesting that they were important factors for AO generation. Further, the transcription factors *FOXF1* and *TCF21* were included, and they may be potential markers for lung fibroblasts.

### iMESs expressing high levels of RSPO2 and RSPO3 directly contributed to iAT2 cell induction

We conducted a validation study using fibroblast-free AOs to determine the presence of candidate cytokines in the RNA-seq analysis data. We previously reported that combining the two inhibitors CHIR99021 and SB431542 (2i) contributed to iAT2 cell induction in a fibroblast-free manner (Yamamoto et al., 2017), and we hypothesized that Wnt ligands and antagonists of transforming growth factor β (TGF-β) family ligands increased in iMESs could substitute for 2i. Because only Wnt/β-catenin seemed important for AT2 cell differentiation (Aros et al., 2021; Frank et al., 2016; Shu et al., 2005), highly expressed canonical Wnt ligands and antagonists of TGF-β family ligands were selected, but WNT5A, WNT5B, and WNT11, which are associated with the non-canonical Wnt signaling pathway, were excluded (Table S3) (Cohen et al., 2012; Hardy et al., 2008; Mikels and Nusse, 2006). HDFs did not contain any canonical Wnt ligands transcribed in the defined list (Table S3). We noted that *RSPO2* and *RSPO3* transcript levels were higher in HFLF- and HDF-iMESs and HFLFs than in HDFs. To test the ability of these candidates to produce AOs in a fibroblast-free environment, we incubated cells in a medium supplemented with CHIR99021/SB431542 (2i), RSPO2/SB431542, RSPO3/SB431542, RSPO2/RSPO3, or RSPO2/RSPO3/SB431542 (Figure S2C). RSPO2/RSPO3/SB431542 increased the SFTPC-GFP^+^/EPCAM^+^ iAT2 cell ratio to a level comparable to that of 2i (Figures S2D and S2E). Next, we selected candidate endogenous genes of TGF-β-ligand antagonists to replace SB431542 (Table S3). We noted that FST, FSTL1, FSTL3, and DCN had high enough TPM values in iMESs to test despite lower expression levels in both HFLF- and HDF-iMESs than in post-3D culture HDFs. Fibroblast-free AOs were formed in a medium supplemented with RSPO2/RSPO3/SB431542, RSPO2/RSPO3/FST, RSPO2/RSPO3/FSTL1, RSPO2/RSPO3/FSTL3, RSPO2/RSPO3/DCN, RSPO2/RSPO3/3F (FST/FSTL1/FSTL3), and RSPO2/RSPO3/4F (FST/FSTL1/FSTL3/DCN). However, the SFTPC-GFP^+^/EPCAM^+^ iAT2 cell ratio did not increase with any factor supplementation (Figures S2F and S2G).

### iMESs expanded iAT2 cells by repeated passages

**Figure 3:**
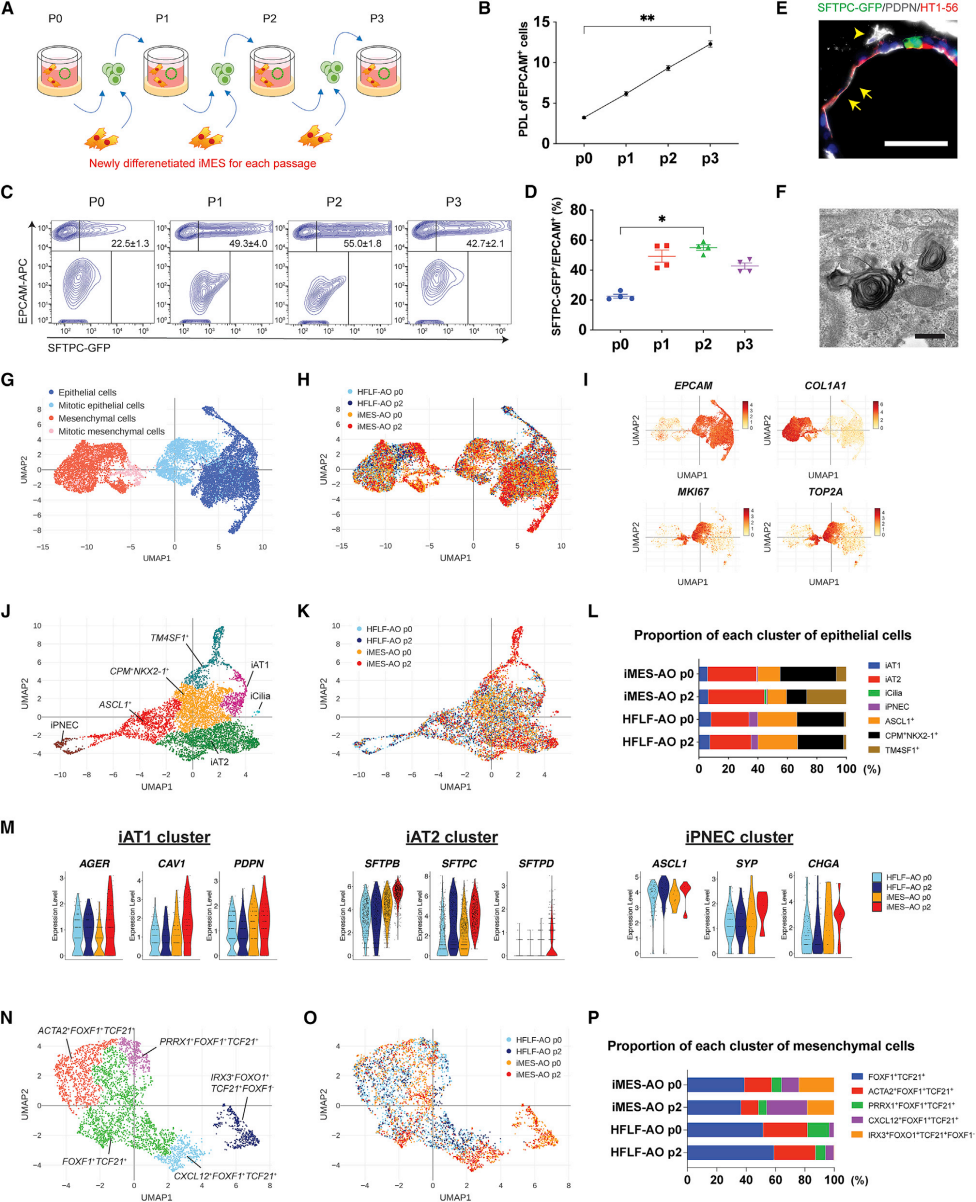
Passage culture of SFTPC-GFP^+^/EPCAM^+^ iAT2 cells in iMES-AOs and single-cell transcriptomics of iMES-AOs and HFLF-AOs (A) Schematic for passage culture of iMES-AOs. SFTPC-GFP^+^/EPCAM^+^ cells were repeatedly passaged with newly differentiated iMESs. (B) PDL of EPCAM^+^ cells in iMES-AOs. Data are presented as mean ± SEM (n = 4, independent experiments). ∗∗p < 0.01 (Dunn’s post hoc test). (C and D) Flow cytometry analysis to evaluate SFTPC-GFP^+^/EPCAM^+^ cell ratio in each passage and their quantification. The iMES-AOs consisted of epithelial cells derived from the SFTPC-GFP reporter iPSC line (B2-3) and HFLF-iMESs. (E) Immunofluorescence staining of AT1 markers and SFTPC-GFP in the iMES-AOs (P2). Scale bars, 50 μm. (F) Transmission electron microscope image of lamella bodies of the iMES-AOs (P2). Scale bar, 500 nm. (G) Graph-based clustering and cell-type annotation of all samples. The iMES-AOs consisted of epithelial cells derived from the SFTPC-GFP reporter iPSC line (B2-3) and 201B7-iMESs. The HFLF-AOs consisted of epithelial cells derived from the SFTPC-GFP reporter iPSC line (B2-3) and HFLFs. (H) A uniform manifold and approximation projection (UMAP) plot showing all 4 samples including HFLF-AOs at P0, HFLF-AOs at P2, iMES-AOs at P0, and iMES-AOs at P2. (I) A UMAP plot of genes that provided the basis for each cluster. (J and K) Graph-based clustering and cell-type annotation of epithelial cells except for mitotic cells. (L) Quantification of each cluster of epithelial cells. (M) Violin plots of the representative genes of each epithelial cluster. (N and O) Graph-based clustering and cell-type annotation of mesenchymal cells except for mitotic cells. (P) Quantification of each cluster of mesenchymal cells. See also Figure S3.

We performed passage culture of SFTPC^+^/EPCAM^+^ iAT2 cells with newly differentiated iMESs prepared for each passage (Figure 3A). The cumulative population doubling level (PDL) of EPCAM^+^ cells increased linearly (Figure 3B). The SFTPC^+^/EPCAM^+^ iAT2 cell ratio immediately increased after a single passage, and it significantly increased from passage 0 (P0) to P2 (p = 0.002) (Figures 3C and 3D). qRT-PCR showed that *ABCA3* and *SLC34A2* (AT2 markers) and *HOPX* (AT1 marker) significantly increased from P0 to P3 (Figure S3A). Expression of other alveolar lineage markers, *SFTPB*, *SFTPD2*, *SFTPA2*, *AGER*, and *AQP5*, were maintained during the passages. Immunofluorescence (IF) staining showed both SFTPC-GFP^+^ iAT2 and PDPN^+^/HT1-56^+^ iAT1 cells in P2-AOs (Figure 3E). We used transmission electron microscopy to visualize the lamellar bodies and specific structures of AT2 cells (Figure 3F).

### scRNA-seq analysis revealed multiple cell types in iMES-AOs and HFLF-AOs

We performed single-cell RNA-seq (scRNA-seq) to compare iMES-AOs (derived from B2-3 lung progenitors and 201B7-iMES) with HFLF-AOs (derived from B2-3 lung progenitors and HFLFs) at both P0 and P2. Epithelial and mesenchymal cells were segregated by expressing high levels of *EPCAM* and *COL1A1*, respectively, and clusters of mitotic cells expressing *MKI67* and *TOP2A* were also identified in both types of cells (Figures 3G–3I). Non-mitotic epithelial cells were re-clustered and annotated based on enriched gene expression profiles. iAT1, iAT2, iPSC-derived ciliary cells (iCilias), iPSC-derived pulmonary neuroendocrine cells (iPNECs), *ASCL1*^*+*^ clusters, and *TM4SF1*^*+*^ clusters were identified (Figures 3J and S3B). iMESs and HFLFs seemed to have different inductive abilities because iAT2 and iCilia were more numerous in iMES-AOs, especially since iCilia was seen only in iMES-AOs at P2 (Figures 3K and 3L). Conversely, iAT1, iPNEC, and *ASCL1*^*+*^ cells were more numerous in HFLF-AOs. However, expression levels of AT1, AT2, and PNEC markers in each cluster tended to be higher in iMES-AOs than in HFLF-AOs (Figure 3M). *TM4SF1*^*+*^ cells were abundant in iMES-AOs at P2 (Figures 3K and 3L). iMESs without mitotic cells were separated into five clusters: *FOXF1*^+^*TCF21*^+^, *ACTA2*^+^*FOXF1*^+^*TCF21*^+^, *PRRX1*^+^*FOXF1*^+^*TCF21*^+^, *CXCL12*^+^*FOXF1*^+^*TCF21*^+^, and *IRX3*^+^*FOXO1*^+^*TCF21*^+^*FOXF1*^−^ (Figure 3N). The representative genes were depicted on violin plots (Figure S3C). *TCF21* was expressed in all clusters, while *FOXF1* was expressed in all but the *IRX3*^+^*FOXO1*^+^*TCF21*^+^*FOXF1*^−^ cluster. *CXCL12*^+^*FOXF1*^+^*TCF21*^+^ cells were abundant in iMES-AOs, and *WT1* was exclusively expressed in iMESs. The *IRX3*^+^*FOXO1*^+^*TCF21*^+^*FOXF1*^−^ cluster consisted almost entirely of iMESs (Figures 3O and 3P). *RSPO2* was expressed considerably more in iMESs, and RSPO3 was expressed in both HFLFs and iMESs (Figure S3C). *WNT2*, *CTHRC1*, and *MYH11* were almost exclusively expressed in HFLFs. *ACTA2*^+^*MYH11*^+^ mature myogenic cells were mostly seen in HFLFs.

### H1N1 and SARS-CoV-2 infection of iMES-AOs induce intrinsic interferon responses

**Figure 4:**
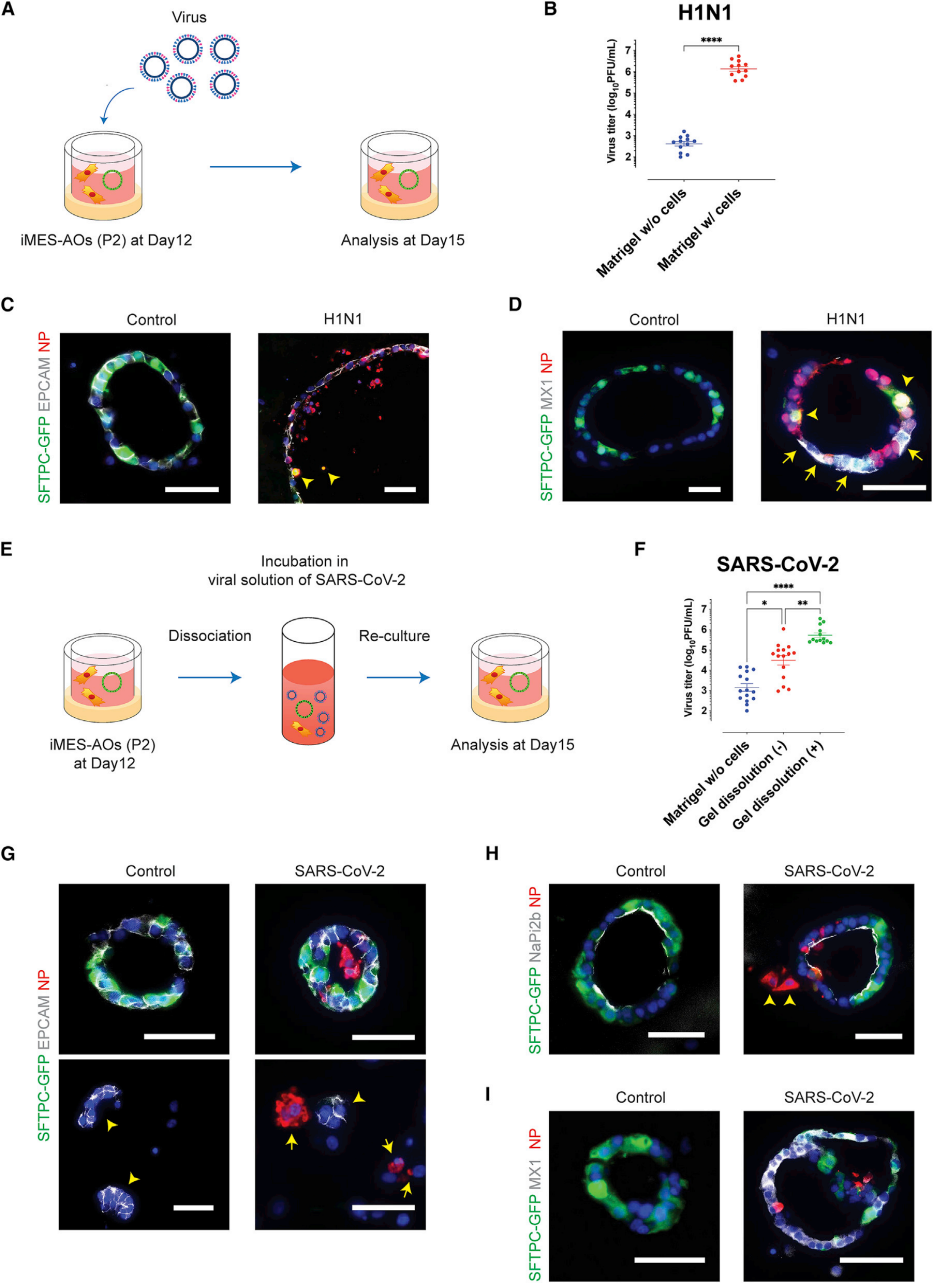
Viral infection models revealed intrinsic interferon responses (A) Schematic for viral infection to iMES-AOs at P2. Viral solution was added to upper and lower chambers, and iMES-AOs were exposed to the virus from days 12–15 after the final passage. The iMES-AOs consisted of epithelial cells derived from the SFTPC-GFP reporter iPSC line (B2-3) and HFLF-iMESs. (B) Viral titer measured by a plaque-forming assay (n = 4 independent experiments with 3 wells each). ∗∗∗∗p < 0.0001 (Mann-Whitney test). (C) Immunofluorescence staining of the nucleoprotein (NP) of influenza A virus, EPCAM, and SFTPC-GFP. Arrowhead: infected SFTPC-GFP^+^ cells. (D) Immunofluorescence staining of NP of influenza A virus, MX1, and SFTPC-GFP. Arrowhead: infected SFTPC-GFP^+^ cells. Arrow: MX1^+^ cells. Scale bars, 50 μm. (E) Schematic for viral infection to iMES-AOs at P2 after dissociation of whole gels. The iMES-AOs consisted of epithelial cells derived from the SFTPC-GFP reporter iPSC line (B2-3) and HFLF-iMESs. (F) Viral titer measured using a plaque-forming assay (n = 4–5 independent experiments with each from 3-4 wells). ∗p < 0.05, ∗∗p < 0.01, ∗∗∗∗p < 0.0001 (Dunn’s post hoc test). (G) Immunofluorescence staining of NP of SARS-CoV-2, EPCAM, and SFTPC-GFP. Arrowhead: fragmented spheroids after dissociation. Arrow: infected EPCAM^−^ iMESs. Scale bars, 50 μm. (H) Immunofluorescence staining of NP of SARS-CoV-2, NaPi2b, and SFTPC-GFP. Arrowhead: infected iMESs. Scale bars, 50 μm. (I) Immunofluorescence staining of NP of WK-521, MX1, and SFTPC-GFP. Scale bars, 50 μm.

We applied iMES-AOs to disease modeling of acute respiratory viral infections. iMES-AOs (P2) on day 12 were infected with H1N1 or SARS-CoV-2 for 3 days and then collected for analysis (Figure 4A). The viral titer was higher in H1N1-infected iMES-AOs than in the no-cell control (6.1 ± 0.1 versus 2.6 ± 0.1, log_10_PFU/mL) (Figure 4B). The nucleoprotein of H1N1 was detected in EPCAM^+^ epithelial cells, and some infected cells, including SFTPC-GFP^+^ iAT2 cells, fell into the lumen (Figure 4C). We observed MX1^+^ cells, showing a type I interferon response induced by H1N1 infection (Figure 4D). In contrast, the nucleocapsid protein of SARS-CoV-2 was not stained in SARS-CoV-2-infected iMES-AOs. We speculated that inaccessibility to the apical inside of AOs interfered with the efficient infection of SARS-CoV-2, while H1N1 could invade AOs from the basolateral side, where sialic acid is present. Thus, we dissociated whole gels of iMES-AOs and then incubated the gels in a viral solution of SARS-CoV-2 for 2 h, as described previously (Mulay et al., 2021). After washing, they were re-cultured in a 3D culture with Matrigel (Figure 4E). Three days later, the viral titer increased compared with both the no-cell control and the previous non-dissociated samples (3.2 ± 0.2 versus 4.5 ± 0.2 versus 5.7 ± 0.1, log_10_PFU/mL) (Figure 4F), suggesting that the virus could approach the apical side of organoids where abundant receptors are present. The nucleocapsid protein of SARS-CoV-2 was also detected (Figure 4G). NaPi2b and the nucleocapsid protein were co-stained, indicating infected AT2 cells (Figure 4H), and MX1^+^ cells indicated that the type I interferon response was induced by SARS-CoV-2 infection (Figure 4I).

## Discussion

In this study, we featured iMESs that efficiently induced SFTPC-GFP^+^/EPCAM^+^ iAT2 cells via epithelial-mesenchymal interactions. Although the iMES transcriptome did not completely match that of HFLFs, our approach to generate human PSC-derived mesenchymal cells that are suited for inducing tissue epithelial cells, such as iAT1 and iAT2 cells, might inform us of the most crucial factors in organogenesis.

Previous studies have reported a method of induction of respiratory mesenchymal cells (Han et al., 2020; Kishimoto et al., 2020). In these studies, NKX6-1 was induced as a respiratory mesenchymal marker with retinoic acid, BMP4, and Hedgehog agonists, followed by a low-dose WNT agonist. Intriguingly, although iMESs do not express *NKX6-1*, we succeeded in expressing SFTPC-GFP in alveolar epithelial cells in iMES-AOs. Indeed, we observed a low *NKX6-1* expression level, even in HFLFs; thus, we speculated that NKX6-1 might not be requisite for inducing AOs. Moreover, a recent study reported the differentiation of mouse PSC-derived lung-specific mesenchymal cells via *TBX4*^+^ state (Alber et al., 2022). Although AT1/AT2 markers, including *Sftpc*, *Ager*, and *Hopx*, were not robustly induced in the study, it was demonstrated that lung progenitor cells expressed early distal lung epithelial markers, such as *Sox9* and *Etv5*, indicating insufficient inducing factors of AT1/AT2 cells. iMES transcriptome analysis and subsequent validation revealed that RSPO2 and RSPO3 expressed in iMESs could promote iAT2 cell induction. This is consistent with a recent report that revealed that RSPO2^+^ mesenchymal cells were adjacent to human fetal lung bud tip progenitors and that RSPO2 might potentially possess a pivotal role in proximal-distal patterning (Hein et al., 2022). The low expression level of *WNT2* was unexpected because Wnt2/2b signaling has been reported to be essential for lung endoderm specification (Goss et al., 2009). However, RSPO2/RSPO3 expressed in iMESs could promote iAT2 cell induction, substituting for the WNT2 expressed in HFLFs for activation of canonical WNT signaling. The lack of canonical Wnt ligand gene expression in HDFs might be one reason for their inability to induce iAT2 cells. On the other hand, it was an unexpected result that *WNT5A*, *FGF7*, and *PDGFRA*, known to be important factors in AT2 cells (Barkauskas et al., 2013; Nabhan et al., 2018; Zepp et al., 2017), appeared to be up-regulated in HDFs, compared with iMESs and HFLFs, although FGF7 was supplemented in the alveolarization medium. In a previous study, we reported that AOs could not develop without mesenchymal cells if CHIR99021, SB431542, and Y27632 were not added (Yamamoto et al., 2017). Therefore, there should be cell-cell interactions between iMESs and alveolar epithelial cells that at least complement the role of SB431542. All in all, further studies are needed to clarify AT2 cell induction down-stream pathways. We also elucidated that iMES-AOs and HFLF-AOs included multiple cell types using scRNA-seq transcriptomics. Expression levels of each lineage marker tended to be higher in iMES-AOs, suggesting that iMESs could induce more mature respiratory epithelial cells compared with HFLFs. *FOXJ1*^*+*^*/RSPH1*^*+*^ iCilia observed in iMES-AOs at P2 co-expressed *SFTPC*; hence, they may be cells that would differentiate to mature multiciliated cells from *SFTPC*^*+*^ distal tip cells. *TM4SF1*^+^ cells were numerous in iMES-AOs at P2. TM4SF1 has been noted as a marker of Wnt-responsive alveolar epithelial progenitor lineage (Zacharias et al., 2018), and further verification is needed on whether *TM4SF1*^+^ cells in iMES-AOs could serve as progenitors. In the analysis of mesenchymal cells, the *IRX3*^+^*FOXO1*^+^*TCF21*^+^*FOXF1*^−^ cluster and the *CXCL12*^+^*FOXF1*^+^*TCF21*^+^ cluster were distinctive in iMESs. Further studies are needed to determine if these clusters have the power to induce respiratory epithelial cells, particularly iCilia that could be induced by iMESs but not by HFLFs.

In conclusion, iMES-AOs may be used to investigate the central mechanism of alveolar differentiation through epithelial-mesenchymal interactions, which would be helpful for disease models, drug screening, and niche reconstruction for *in vivo* lung regeneration in the future.

### Limitations of the study

It is advantageous that the ratio of SFTPC-GFP^+^/EPCAM^+^ cells increased until P3 in iMES-AOs, but in this study, we did not validate the phenotypes of iMES-AOs in long-term passages. We applied the iMES-AO platform to the H1N1 and SARS-CoV-2 infection models, and iMES-AOs were analyzed at 72 h post-infection because the highest viral titers were observed in the 72 h sample compared with the 24 and 48 h samples (data not shown). Given that the lineage markers can disappear after infection, early evaluation, such as at 24 h post-infection, may clarify initial infection target cell types.

## STAR★Methods

### Key resources table

**Table:** 

REAGENT or RESOURCE	SOURCE	IDENTIFIER
**Antibodies**		

Anti-T -Alexa Fluor 488	RD systems	Cat# IC2085G; RRID: not listed.
Anti-NCAM -Alexa Fluor 647	BioLegend	Cat# 362513; RRID: AB_2564086
Anti-PDGFRA -Alexa Fluor 647	BD Biosciences	Cat# 562798; RRID: AB_2737803
Anti-KDR -BV421	BioLegend	Cat# 393009; RRID: AB_2832739
Anti-Vimentin -Alexa Fluor 647	Novus Biologicals	Cat# NBP1-97670AF647; RRID: not listed.
Anti-CD90 -BV421	BioLegend	Cat# 328121; RRID: AB_10933261
Anti-NANOG -Alexa Fluor 488	BD Biosciences	Cat# 560791; RRID: AB_1937305
Anti-OCT3/4 -Alexa Fluor 647	BD Biosciences	Cat# 560329; RRID: AB_1645318
Anti-FOXA2 -PE	BD Biosciences	Cat# 561589; RRID: AB_10716057
Anti-SOX17 -Alexa Fluor 647	BD Biosciences	Cat# 562594; RRID: AB_2737670
Anti-Nestin -BV421	BioLegend	Cat# 656808; RRID: AB_2566634
Anti-PAX6 -Alexa Fluor 488	BD Biosciences	Cat# 561664; RRID: AB_10895587
Anti-EPCAM -FITC	Miltenyi Biotec	Cat# 130-080-301; RRID: AB_244192
Anti-EPCAM -APC	Miltenyi Biotec	Cat# 130-113-260; RRID: AB_2726061
Anti-EPCAM -BV421	BD Biosciences	Cat# 563180; RRID: AB_2738050
Isotype control mouse IgG2a -Alexa Fluor 647	BioLegend	Cat# 400239; RRID: not listed.
Isotype control mouse IgG1 -BV421	BioLegend	Cat# 400157; RRID: AB_10897939
Isotype control mouse IgG1 -Alexa Fluor 647	BioLegend	Cat# 400130; RRID: AB_2800436
Isotype control mouse IgG1 -Alexa Fluor 488	BioLegend	Cat# 400132; RRID: not listed.
Isotype control mouse IgG1 -PE	Beckman Coulter	Cat# A07796; RRID: AB_2832963
Isotype control mouse IgG2a -Alexa Fluor 488	BD Biosciences	Cat# 565362; RRID: AB_2869664
Propidium Iodide	Nacalai tesque	Cat# 29037–76
Anti-EPCAM	Santa Cruz Biotechnology	Cat# sc-66020/EBA-1; RRID: AB_2098654
Anti-mouse IgG -MicroBeads	Miltenyi Biotec	Cat# 130-048-401; RRID: AB_244360
Anti-CPM	Wako	Cat# 014–27501; RRID: AB_2801482
Anti-mouse IgG Alexa Fluor 647	Thermo Fisher Scientific	Cat# A-31571; RRID: AB_162542
Anti-E-Cadherin	eBiosience	Cat# 14–3249; RRID: AB_1210459
Anti-Vimentin	CST	Cat# 49636; RRID: AB_2799363
Anti-FOXF1	RD systems	Cat# AF4798; RRID: AB_2105588
Anti-ProSPC	Seven Hills	Cat# WRAB-9337; RRID: AB_2335890
Anti-pro and mature SPB	Abcam	Cat# ab40876; RRID: AB_778186
Anti-matureSPC	Seven Hills	Cat# WRAB-76694; RRID: not listed.
Anti-ABCA3	Seven Hills	Cat# WMAB-17G524; RRID: not listed.
Anti-HT1-56	Terrace	Cat# TB-29AHT1-56; RRID: AB_2847898
Anti-PDPN -APC	eBioscience	Cat# 17-9381-42; RRID: AB_10801951
Anti-GFP	Aves Labs	Cat# GFP-1020; RRID: AB_10000240
Anti-EPCAM	RD systems	Cat# AF960; RRID: AB_355745
Anti-InfluenzaA H3N2 NP	Sino Biological	Cat# 40208-RP01; RRID: not listed.
Anti-SARS-CoV/SARS-CoV-2 NP	GeneTex	Cat# GTX632269; RRID: AB_2888304
Anti-MX1	RD systems	Cat# AF7946; RRID: not listed.
Anti-NaPi2b	kindly provided by Dr. Gerd Ritter (MX35)	N/A
Anti-rat IgG Alexa Fluor 488	Thermo Fisher Scientific	Cat# A-21208; RRID: AB_2535794
Anti-mouse IgG Alexa Fluor 546	Thermo Fisher Scientific	Cat# A-10036; RRID: AB_2534012
Anti-goat IgG Alexa Fluor 647	Thermo Fisher Scientific	Cat# A-21447; RRID: AB_2535864
Anti-rabbit IgG Alexa Fluor 647	Thermo Fisher Scientific	Cat# A-31573; RRID: AB_2536183
Anti-chicken IgY Alexa Fluor 488	Jackson ImmunoResearch	Cat# 703-546-155; RRID: AB_2340376
Anti-rabbit IgG Cy3	Jackson ImmunoResearch	Cat# 711-165-152; RRID: AB_2307443
Anti-mouse IgG Alexa Fluor 488	Thermo Fisher Scientific	Cat# A-21202; RRID: AB_141607
Anti-mouse IgG1 Cy3	Jackson ImmunoResearch	Cat# 115-165-205; RRID: AB_2338694

**Bacterial and virus strains**		

Influenza A H1N1 A/Narita/1/2009	National institute of infectious diseases	N/A
SARS-CoV-2 WK521	National institute of infectious diseases	N/A

**Biological samples**		

Nothing		

**Chemicals, peptides, and recombinant proteins**		

PBS	Nacalai tesque	14249–24
Geltrex	Thermo Fisher Scientific	A1413302
iMatrix-511 silk	Nippi	892021
Matrigel Growth Factor Reduced Basement Membrane Matrix	Corning	354230
Essential 8	Thermo Scientific	A1517001
STEMdiff™ Trilineage Differentiation Kit	STEMCELL	ST-05230
mTeSR plus	STEMCELL	ST-05825/ST-100-0276
Penicillin–streptomycin	Thermo Fisher Scientific	15140–163
Y-27632	LCL labolatories	LCL-Y-5301-250
StemPro™-34	Thermo Fisher Scientific	10639011
Glutamax	Thermo Scientific	35050–061
ActivinA	API	GF-001-050L
BMP4	RD systems	314-BP-01M
CHIR99021	AXON Medchem	AXN-AXON1386-25
KGF	Peprotech	100-19-250UG
bFGF	KAC	KHFG001
FGF10	Peprotech	100–26
RPMI 1640	Nacalai tesque	30264–56
DMEM/F12 plus Glutamax	Thermo Fisher Scientific	10565–042
Ham's F12	Wako	087–08355
DMEM with high glucose	Nacalai tesque	08459–64
Fetal bovine serum	Nichirei	175012
B27 supplement	Thermo Fisher Scientific	17504–001
L-ascorbic acid	Sigma	A4403
Monothioglycerol	Wako	195–15791
Bovine Serum Albumin Fraction V Solution (7.5%)	Thermo Fisher Scientific	15260–037
HEPES 1M solution	Life Sciences	SH30237-01
CaCl2	Wako	036–19731
ITS premix	Corning	354350
Dexamethasone	Sigma-Aldrich	D4902-25MG
IBMX	Wako	099–03411
8-Br-cAMP	LIFE SCIENCE INSTITUTE	B007-100
R-spondin 2	RD systems	3266-RS-025/CF
R-spondin 3	RD systems	3500-RS-025/CF
FST	RD systems	4889-FN-025/CF
FSTL1	RD systems	1694-FN-050
FSTL3	RD systems	1288-F3-025/CF
DCN	RD systems	143-DE-100
Sodium butyrate	Wako	193–01522
SB431542	Wako	198–16543
Noggin	RD systems	6057-NG-01M
Retinoic acid	Sigma-Aldrich	R2625
DAPT	Wako	049–33583

**Critical commercial assays**		

Fixation and Permeabilization Solution	BD Biosciences	554722
Perm/Wash Buffer	BD Biosciences	554723
PureLink RNA mini kit	Thermo Fisher Scientific	12183020
Rneasy micro kit	Quiagen	74004
RNaseOUT™ Recombinant Ribonuclease Inhibitor	Thermo Fisher Scientific	10777019
SuperScript™ III Reverse Transcriptase	Thermo Fisher Scientific	18080044
Power SYBR Green PCR Master Mix	Thermo Fisher Scientific	4368708
Human iPS Cell Generation™ Episomal Vector Mix	Takara	3673
Chromium Next GEM Single Cell 3ʹ GEM, Library & Gel Bead Kit v3.1	10× Genomics	PN-1000128
Chromium Next GEM Single Cell 3ʹ Kit v3.1	10× Genomics	PN-1000269
Chromium Next GEM Chip G Single Cell Kit	10× Genomics	PN-1000127
Single Index Kit T Set A	10× Genomics	PN-1000213
Dual Index Kit TT Set A	10× Genomics	PN-1000215

**Deposited data**		

Bulk RNA-seq data	This paper	Access number GEO:188822
scRNA-seq data	This paper	Access number GEO:188823

**Experimental models: Cell lines**		

Human healthy donor iPSC line (201B7)	Takahashi et al.	https://www.cira.kyoto-u.ac.jp
201B7 iPSC line targeted with SFTPC-GFP (B2-3)	Gotoh Lab	Gotoh Lab
Human normal donor iPSC line (604A1)	Okita et al.	https://www.cira.kyoto-u.ac.jp
HFLF	DV Biologics	PP002-F-1349 (Discontinued)
HDF (TIG120)	Kondo et al.	https://cellbank.nibiohn.go.jp
HFLF iPSC (HFA)	This paper	N/A
HDF (TIG120) iPSC (GC23)	This paper	N/A
MDCK	National institute of infectious diseases	N/A
VeroE6/TMPRSS2	National institute of infectious diseases	N/A

**Oligonucleotides**		

Primers for qRT-PCR are listed in Table S3.	N/A	N/A

**Software and algorithms**		

FlowJo	FlowJo, LLC	N/A
FIJI	N/A	https://imagej.net
Prism 9 for Mac OSX	GraphPad	N/A
fastp 0.20.1	N/A	https://github.com/OpenGene/fastp#install-with-bioconda
SortMeRna 2.1b	N/A	https://github.com/biocore/sortmerna
STAR 2.7.6a	N/A	https://github.com/alexdobin/STAR
RSEM 1.3.3	N/A	https://github.com/deweylab/RSEM
R 4.1.1	CRAN	http://www.R-project.org
tximport 1.20.0	N/A	https://github.com/mikelove/tximport
DESeq2 1.32.0	N/A	https://github.com/mikelove/DESeq2
clusterProfiler 4.0.5	N/A	https://github.com/YuLab-SMU/clusterProfiler
org.Hs.eg.db 3.13.0	N/A	https://anaconda.org/bioconda/bioconductor-org.hs.eg.db
Cell Ranger 4.0.0	10× Genomics	https://www.10xgenomics.com
Seurat 4.0.3	N/A	https://github.com/satijalab/seurat
r-plotly 4.9.4.1	N/A	https://anaconda.org/conda-forge/r-plotly

**Other**		

Accutase	Innovative Cell Technologies	#AT-104-500
2.5g/L-Trypsin/1mmol/L-EDTA Solution	Nacalai tesque	32777–15
TrypLE™ Select Enzyme (1X)	Thermo Fisher Scientific	12563029
DispaseⅡ	Wako	383–02281
Cell Culture Insert 0.4μm pore size 24well format	Corning	353095
Cell Culture Insert 0.4μm pore size 12well format	Corning	353180
MACS LS column	Miltenyi Biotec	130-042-401
MACS LD column	Miltenyi Biotec	130-042-901
Elplasia 96-well plate	Corning	4446
Poly(2-hydroxyethyl methacrylate)	Sigma-Aldrich	192066
Donkey Serum	EMD-Millipore	S30-100ML

### Resource availability

#### Lead contact

Further information and requests for resources and reagents should be directed to and will be fulfilled by the lead contact, Shimpei Gotoh (gotoh.shimpei.5m@kyoto-u.ac.jp).

#### Materials availability

201B7 and 604A1 were obtained from CiRA at Kyoto University. TIG120 was obtained from JCRB (JCRB0542). B2-3, HFA and GC23 are available from the lead contact upon request. HFLF has been discontinued (DV Biologics) but is available from the lead contact upon request for use in academia.

### Experimental model and subject details

#### Generation of human iPSCs

HFLF-iPSCs (HFA) were established from HFLF (17.5 weeks of gestation; DV Biologics; PP002-F-1349, lot 121109VA). HFLF (1 × 10^6^ cells) were transfected with human iPSC generation episomal vector mix containing cDNA of OCT3/4, SOX2, KLF4, L-MYC, LIN28, mp53-DD, and EBNA1 (Takara, 3673). Transfected cells (5 × 10^4^ cells) were seeded on a well of a 6-well plate with 10% fetal bovine serum/Dulbecco’s Modified Eagle Medium (FBS/DMEM). The medium was replaced with 10% FBS/DMEM on days 1, 3, and 5, and switched to StemFit AK02N (Ajinomoto, AJ100) on day 6. The generated iPSC colonies were picked up and replated to a well of a 12-well plate with StemFit AK02N and iMatrix-511 silk (Nippi, 892021) (0.25 μg/cm^2^). The generated iPSCs were maintained in StemFit AK02N. The medium was switched to mTeSR Plus (STEMCELL technologies, ST-05825 or ST-100-0276) after several passages, and then used for subsequent differentiation experiments. HDF-iPSCs (GC23) were established from HDF (TIG120) in a feeder-dependent manner by using episomal vectors (OCT3/4, SOX2, KLF4, L-MYC, LIN28, short hairpin RNA for p53), as described previously (Korogi et al., 2019). After expansion and stocking, HDF-iPSCs were maintained in a feeder-free manner with mTeSR Plus medium prior to differentiation. Trilineage differentiation of HFA- and GC23-iPSCs into endoderm, mesoderm, and ectoderm was validated using a STEMdiff™ Trilineage Differentiation kit (STEMCELL, ST-05230), according to the manufacturer’s protocol.

#### Maintenance of iPSCs

Stock vials of SFTPC-GFP reporter iPSCs (B2-3), 201B7, or 604A1 were thawed with the prewarmed Essential 8 medium (Thermo Fischer Scientific, A1517001). Cell suspensions were centrifuged with a mini centrifuge for 1 min. Cells resuspended in 4 mL of Essential 8 medium supplemented with 10 μM Y27632 and 50 U/mL penicillin/streptomycin (ThermoFisher, 15140–163) were seeded on a 6-cm dish coated with Geltrex (Thermo Fischer Scientific, A1413201). Cells were passaged to be 80–90% confluent at an appropriate split ratio (1:4–1:6). The medium was changed daily. For HFLF-iPSCs and HDF-iPSCs, stock vials were thawed with prewarmed mTeSR Plus medium. Cell suspensions were centrifuged with a mini centrifuge for 1 min. Resuspended cells in 4 mL of mTeSR Plus medium supplemented with 10 μM Y27632, iMatrix-511 silk (0.25 μg/cm^2^), and 50 U/mL penicillin/streptomycin were seeded on the 6-cm dish. Cells were passaged to be 80–90% confluent at an appropriate split ratio (1:10–1:50). The next day, the medium was changed to remove Y27632 and subsequently changed every two days.

### Method details

#### Induction of iMES

Undifferentiated human iPSCs were washed in D-PBS and incubated in Accutase (Innovative Cell Technologies, AT-104) at 37°C for 20 min to dissociate the cells into single cells. After neutralizing Accutase by adding one volume of mTeSR Plus, the cells were centrifuged with a mini centrifuge for 1 min, and the supernatant was removed. Then, the recovered single-cell suspension was seeded onto a 6 well-plate at a density of 8–15 × 10^4^ cells/well in mTeSR Plus containing iMatrix-511 silk (0.25 μg/cm^2^) and 10 μM Y-27632 (LC Laboratories, Y-5301) on day −1. On day 0, the medium was replaced with StemPro™-34 (Thermo Fisher Scientific, 10639011) supplemented with 15 ng/mL Activin A (API, GF-001), 50 ng/mL BMP4 (RD systems, 314-BP), 1.5 μM CHIR99021 (Axon Medchem, Axon1386) as an activator of canonical Wnt signaling, Glutamax (Thermo Fisher Scientific, 35050061), and 50 U/mL penicillin/streptomycin (Table S1), and the medium was replaced on day 2. On day 3, the medium was switched to StemPro™-34 supplemented with 3 ng/mL Activin A, 10 ng/mL KGF (Prospec, CYT-219), 25 ng/mL BMP4, 10 ng/mL bFGF (DS Pharma Biomedical, KHFGF001), 10 ng/mL FGF10 (Peprotech, 100-26), Glutamax (×100), and 50 U/mL penicillin/streptomycin and replaced on day 5. On day 7, the cells were detached with TrypLE Select Enzyme (Thermo Fischer Scientific,12563029) at 37 °C for 10 min. The single-cell suspension was washed with 2% FBS/DMEM and labeled with anti-EPCAM antibody (Santa Cruz Biotechnology, sc-66020/EBA-1) by incubating the cells in the antibody solution at 1 μL in 100 μL of 1% bovine serum albumin (BSA)/PBS per million cells at 25 °C for 20 min. Then, EPCAM^−^ cells were negatively isolated by Magnetic-activated cell sorting (MACS) using anti-mouse IgG microbeads (Miltenyi Biotec, 130-048-401) as the secondary antibody and an LD column (Miltenyi Biotec, 130-042-901), according to the manufacturer’s instructions. The recovered mesenchymal cells were used as the iMES.

#### Induction of NKX2-1^+^ lung progenitor cells

Human iPSCs were differentiated into lung progenitor cells, as previously described (Gotoh et al., 2014; Konishi et al., 2016; Yamamoto et al., 2017). In brief, undifferentiated human iPSCs were differentiated into definitive endodermal cells on Geltrex-coated plates in RPMI1640 (Nacalai Tesque, 30264–56) containing 100 ng/mL activin A, 1 μM CHIR99021, 2% B27 supplement (ThermoFisher, 17504–001), and 50 U/mL penicillin/streptomycin. The medium was replaced every two days. Y-27632 was supplemented on day 0, and sodium butyrate (Wako, 193–01522) was added on days 1, 2, and 4. During days 6–10, the definitive endodermal cells were cultured in the anteriorization medium, followed by switching to the ventralization medium containing CHIR99021 (3 μM), BMP4 (20 ng/mL) and adjusted doses of ATRA (Sigma-Aldrich, R2625) (Table S2) on day 10. The optimized concentration of ATRA for B2-3 iPSCs was 0.05–0.5 μM. During days 14–21, the cells were cultured in CFKD preconditioning medium (Table S2). On day 21, NKX2-1^+^ lung progenitor cells were isolated using mouse anti-human CPM (Wako, 014–27501) and anti-mouse IgG-Alexa647 (Thermo Fischer Scientific, A-31571) to gate CPM^high^ cells, as previously reported (Yamamoto et al., 2017). The antibodies used are listed in KEY RESOURCES TABLE.

#### AOs formation in a 3D culture and passage of SFTPC^+^ cells

AOs were generated as previously described (Korogi et al., 2019; Yamamoto et al., 2017). A total of 1.0 × 10^4^ CPM^high^ cells and 5.0 × 10^5^ HFLF, TIG120 or iMES were mixed in 100 μL of the alveolarization medium (Table S2) supplemented with Y-27632 (10 μM) and 100 μL of Matrigel (Corning, 354230) and placed on a 12-well cell culture insert (Corning, 353180). The medium in the lower chamber was changed every two days. HFLF were cultured in DMEM (Nacalai Tesque, 08459–64) supplemented with 10% FBS and used at 10 passages. TIG120 cells were cultured in MEM medium (Nacalai Tesque, 21442–25) supplemented with 10% FBS and used within 30 PDL. The whole gels containing AOs were collected in a 15 mL tube and 0.1% Trypsin-EDTA was added. Fragmented gels were gently pipetted and incubated at 37°C for 6 min. Samples were gently resuspended and incubated at 37 °C for an additional 10 min. After neutralizing 0.1% Trypsin-EDTA by 2%FBS, samples were washed twice in 1% BSA/PBS, and immunostained with anti-EPCAM-APC (Miltenyi Biotec, 130-113-263) antibodies. SFTPC-GFP^+^/EPCAM^+^ cells were recovered using flow cytometry. For passage culture, a total of 1.0 × 10^4^ SFTPC-GFP^+^/EPCAM^+^ cells and 5.0 × 10^5^ iMES were mixed in 100 μL of the alveolarization medium supplemented with Y-27632 (10 μM) and 100 μL of Matrigel and placed on a 12-well cell culture insert. Passage was performed every 2 weeks. The antibodies used are shown in KEY RESOURCES TABLE.

#### Validation study of canonical Wnt ligands and TGFβ-inhibitors using fibroblast-free AOs

CPM^+^ lung progenitor cells on day 21 were isolated by MACS using anti-mouse IgG microbeads as the secondary antibody and an LS column (Miltenyi Biotec, 130-042-401), according to the manufacturer’s instructions. A total of 2.0 × 10^5^ of CPM^+^ lung progenitor cells were suspended in 200 μL of alveolarization medium supplemented with each factor (10 μM Y-27632, 3 μM CHIR99021, 200 ng/mL RSPO2 (RD systems, 3266-RS-025/CF), 200 ng/mL RSPO3 (RD systems, 3500-RS-025/CF), 10 μM SB431542 (Wako, 198–16543) as an inhibitor of TGF-β signaling, 200 ng/mL FST (RD systems, 4889-FN-025/CF), 200 ng/mL FSTL1 (RD systems, 1694-FN-050), 200 ng/mL FSTL3 (RD systems, 1288-F3-025/CF), and 200 ng/mL DCN (RD systems, 143-DE-100)) and seeded onto 96 well plates (Corning, 4446) coated with poly (2-hydroxyethyl methacrylate) (Sigma-Aldrich, 192066) to form cell aggregates. The cell aggregates were centrifuged with a mini centrifuge for 1 min after incubation for 24 h at 37°C and 5% CO_2_. The cell aggregate pellets were gently resuspended in 20 μL of precooled Matrigel and placed into a well of a 24-well cell culture plate (Greiner Bio-One, 662160). After incubation for 20 min at 37°C, 500 μL of alveolarization medium supplemented with each factor was added to the Matrigel-embedded cell aggregates and replaced every two days. On day 5, cells were dissociated with 0.1% trypsin-EDTA at 37°C for 15 min. After neutralizing 0.1% Trypsin-EDTA by 2%FBS, samples were washed twice in 1% BSA/PBS and immunostained with anti-EPCAM-APC antibodies. The ratio of SFTPC-GFP^+^/EPCAM^+^ cells in each condition was evaluated using flow cytometry.

#### Viruses

Influenza A virus A/JP/Narita/1/2009 (H1N1; National Institute of Infectious Diseases) was propagated in MDCK cells with trypsin. MDCK cells were cultured in DMEM. The medium was centrifuged at 1400 × *g* for 10 min, and the supernatant was stored at −80°C. The SARS-CoV-2 strain WK-521 (National Institute of Infectious Diseases) was grown in VeroE6 cells expressing human transmembrane serine protease TMPRSS2 (VeroE6/TMPRSS2; JCRB Cell Bank). VeroE6/TMPRSS2 cells were cultured for virus propagation in DMEM supplemented with 2% FBS and 1 mg/mL G418 (Roche, 4727878001). The supernatant recovered from inoculated cell culture was centrifuged at 1400 × *g* for 10 min, and the supernatant was stored at −80°C. All work with SARS-CoV-2 was performed in the biosafety level 3 facility of the National Institute of Infectious Diseases and Kitasato University.

#### Direct infection of influenza A and SARS-CoV-2 viruses of matrigel-embedded organoids

iMES-AOs at P2, consisting of alveolar epithelial cells differentiated from an SFTPC-GFP reporter iPSC line (B2-3) and HFLF-iMES, were prepared in a 24-well format. A total of 5.0 × 10^3^ SFTPC-GFP^+^/EPCAM^+^ cells sorted from iMES-AOs at P1 and 2.5 × 10^5^ iMES were mixed in 50 μL of the alveolarization medium supplemented with Y-27632 (10 μM) and 50 μL of Matrigel and then placed on a 24-well cell culture insert (Corning, 353095) on day 0. On day 12, each viral solution of influenza A H1N1 A/Narita/1/2009 or SARS-CoV-2 WK-521 was added to the medium of the upper and lower chambers at 10^5^ PFU for virus infection. The virus was added to Matrigel without cells and evaluated as a negative control for viral titer measurements. Non-infected controls were prepared for IF staining. iMES-AOs were cultured at 37 °C and 5% CO_2_. Three days post-infection, whole gels were collected for viral titer measurement and IF staining.

#### SARS-CoV-2 WK-521 infection post organoid dissociation

Prior to infection, 500 μg/mL Dispase II (Wako, 383–02281) was added to the upper and lower chambers and incubated for 1 h at 37°C to mildly dissolve iMES-AOs-embedded Matrigel. Whole gels were collected, gently suspended, and washed three times with 1% BSA/PBS. Then, the cells were mixed with the viral solution of SARS-CoV-2 with 10^5^ PFU in 100 μL and incubated at 37°C for 2 h. After washing three times, cells were suspended in 50 μL alveolarization medium, mixed with an equal volume of Matrigel, and seeded onto a cell culture insert of 24-well format. iMES-AOs were cultured at 37°C and 5% CO_2_. Three days post-infection, whole gels were collected for virus titration and IF staining.

#### Flow cytometry

The single-cell suspension was washed with 1% BSA/PBS and immunostained with primary antibodies at 4°C for 15 min. After being washed twice with 1% BSA/PBS, the cells were stained with secondary antibodies at 4°C for 15 min. After being washed twice with 1% BSA/PBS, the cells were stained with propidium iodide (Nacalai Tesque, 29037–76). For intracellular staining, single-cell suspensions were fixed by fixation and permeabilization solution (BD Biosciences, 554722) at 25°C for 20 min. After washing twice with Perm/Wash Buffer (BD Biosciences, 554723), the cells were immunostained with primary antibodies at 4°C for 30 min. After washing twice with Perm/Wash Buffer, the cells were stained with secondary antibodies at 4°C for 30 min. After washing twice with 1% BSA/PBS, the cells were prepared in 1% BSA/PBS without propidium iodide for flow cytometry analysis using Melody (BD Biosciences). The antibodies used are listed in KEY RESOURCES TABLE.

#### RNA-seq analysis

Total RNA was extracted using an RNeasy Micro kit (Qiagen, 74004) according to the manufacturer’s protocol. The libraries were prepared using the TruSeq Stranded mRNA Library Prep kit (Illumina), then they were sequenced using a NovaSeq 6000 (Illumina) platform with 100-bp paired-end reads. FASTQ raw data were trimmed using fastp 0.20.1 (Chen et al., 2018) and then rRNA, tRNA, snRNA, snoRNA, Mt_rRNA, and Mt_tRNA were excluded using SortMeRna 2.1b (Kopylova et al., 2012). They were aligned to GRCh38 using STAR 2.7.6a (Dobin et al., 2013). Read counts and transcripts per million (TPM) were calculated using RSEM 1.3.3 (Li and Dewey, 2011). The data were imported to R 4.1.1 using tximport 1.20.0 (Soneson et al., 2015), and low-expression genes with average read counts among the data set samples under 1 were excluded for downstream analyses. DESeq2 1.32.0 (Love et al., 2014) was used to identify DEGs. Enrichment analysis for GO of biological processes was performed using clusterProfiler 4.0.5 (Wu et al., 2021) and org.Hs.eg.db 3.13.0.

#### scRNA-seq analysis

A single-cell suspension was prepared via enzymatic dissociation. Whole gels were gently suspended in 0.1% Trypsin-EDTA. Fragmented gels were gently pipetted and incubated at 37 °C for 6 min. Samples were suspended gently again and incubated at 37°C for 10 min, washed in alveolarization medium containing 10 μM Y-27632 twice, and filtered through a 40-μm strainer. Single-cell RNA libraries were prepared using a 10× Genomics Chromium device according to the manufacturer’s protocols (Single Cell 3′ Reagent Kits v3.1). The libraries were sequenced using NovaSeq 6000 (Illumina). Reads were mapped to GRCh38, and count matrices were generated using the Cell Ranger (10× Genomics). Processing of the single-cell data was conducted with Seurat 4.0.5 (Hao et al., 2021). In brief, cells expressing mitochondrial genes accounting for >20% and <1.5% were removed to exclude dead and low-quality cells. In addition, outliers of UMI and expressed genes were also removed to exclude doublet and low-quality cells. Then, the UMI count was normalized using SCTransform. Principal component (PC) analysis (PCA) was conducted using the Seurat function RunPCA and embedded in UMAP using the Seurat function RunUMAP on 20 PCs at a resolution of 0.4 (Figures 3G and 3H). UMAP plots were visualized using Plotly 4.9.4.1, and violin plots were drawn using Seurat. Cell-type definition was performed according to expression of representative genes as follows; clusters 1, 2, 3, 9, and 11 were annotated as epithelial cells based on the high expression of *EPCAM*. Clusters 5, 6, and 7 were annotated as mitotic epithelial cells based on the high expression of *EPCAM*, *MKI67*, and *TOP2A*. Clusters 0, 4, and 10 were annotated as mesenchymal cells based on the high expression of *COL1A1*. Clusters 8 was annotated as mitotic mesenchymal cells based on their high expression of *COL1A1*, *MKI67*, and *TOP2A*. The re-clustering of epithelial cells, except for mitotic epithelial cells, was performed. PCA was conducted using the Seurat function RunPCA and embedded in UMAP using the Seurat function RunUMAP on 25 PCs at a resolution of 0.6 (Figures 3J and 3K). Cell-type definition was performed according to expression of representative genes as follows; clusters 9 and 20 were annotated as iAT1 on the high expression of *AGER and CAV1*. Clusters 2, 4, 5, 7, 11, and 15 were annotated as iAT2 based on their high expression of *SFTPB* and *SFTPC*. Cluster 23 showed high expression levels of *FOXJ1*, *RSPH1*, and *SFTPC*, indicative of SFTPC^+^ distal tip cells differentiating into ciliary cells. Cluster 21 and 22 were annotated as iPNEC based on their high expression of *ASCL1*, *SYP* and *CHGA*. The other epithelial clusters were annotated as follows: clusters 1, 3, 6, 8, and 10, *CPM*^+^*NKX2-1*^+^ cells; clusters 0, 12, 14, 17, and 18, *ASCL1*^*+*^ cells; clusters 13, 16, and 19, *TM4SF1*^*+*^ cells. Re-clustering of mesenchymal cells, except for mitotic mesenchymal cells, was performed. PCA was conducted using the Seurat function RunPCA and embedded in UMAP using the Seurat function RunUMAP on 15 PCs at a resolution of 0.7 (Figures 3N and 3O). Cell-type definition was performed according to expression of representative genes as follows; clusters 2, 5, 6, 7, 9, 10, 14, 15 and 16, *FOXF1*^+^*TCF21*^+^ cells; clusters 0, 1, 8 and 17, *ACTA2*^+^*FOXF1*^+^*TCF21*^+^ cells; clusters 11 and 12, *PRRX1*^+^*FOXF1*^+^*TCF21*^+^ cells; clusters 3 and 13, *CXCL12*^+^*FOXF1*^+^*TCF21*^+^ cells; clusters 4, 18 and 19, *IRX3*^+^*FOXO1*^+^*TCF21*^+^*FOXF1*^-^ cells.

#### qRT-PCR

Total RNA was extracted using a PureLink RNA Mini kit (Thermo Fisher Scientific, 12183020). cDNA was prepared from 80 ng of total RNA per sample with SuperScript III reverse transcriptase (Thermo Fisher Scientific, 18080044), amplified using Power SYBR Green PCR Master Mix (Applied Biosystems, 4368708), and quantified using QuantStudio 3 (Applied Biosystems). Gene expression was normalized to β-actin expression levels. The relative gene expression of mesenchymal markers was compared to 201B7 on day 0 (Figure S1A). Exogenous control RNA of human fetal lung at 17, 18, and 22 weeks of gestation was used (Agilent Technologies; #540177, lot 0006055802) to calculate relative AO gene expression (Figures S1D and S3A). The primers used in this study are listed in Table S4.

#### IF staining

Two-dimensional culture was fixed with 4% paraformaldehyde/PBS at 25°C for 15 min, permeabilized with 0.2% Triton X-100/PBS at 25°C for 15 min, and blocked using PBS containing 5% normal donkey serum (EMD-Millipore, S30-100ML). The samples were then immunostained with primary and secondary antibodies. AOs were fixed with 4% paraformaldehyde/PBS at 25°C for 20 min and incubated in 30% sucrose/PBS at 4°C overnight. They were then embedded in the OCT compound (Sakura Finetek, 4583) and frozen in liquid nitrogen. The frozen organoids were sliced into 10-μm-thick sections on slides, permeabilized, and blocked as described above. They were then immunostained with primary antibodies overnight and with secondary antibodies for 1 h. Hoechst-33342 (Dojindo, H342) was added to the secondary antibody solution to label the nuclei. The antibodies used in this study are listed in KEY RESOURCES TABLE. Images were captured using a BZ-X710 microscope (Keyence).

#### Electron microscopy

Whole gels were incubated in a fixative solution consisting of 2.5% glutaraldehyde, 4% paraformaldehyde, 1% tannic acid (Koso Chemical), and 0.1 M phosphate buffer (pH 7.4) at 4°C overnight. The next day, the fixative solution was changed to one without tannic acid. After three washes in 0.1 M phosphate buffer (pH 7.4) for 20 min, the samples were fixed in 1% osmium tetroxide (Nacalai Tesque, 25727–01) for 2 h and gradually dehydrated and embedded in pure epon as previously described (Konishi et al., 2016; Sone et al., 2021). Ultrathin sections were stained with uranyl acetate and lead citrate and analyzed using transmission electron microscopy (JEOL; JEM-1400).

### Quantification and statistical analysis

All error bars indicate the SEM. The quantified data represent the findings of three or more independent experiments. Statistical analyses of qRT-PCR and flow cytometry were performed using the Prism 9 software program (GraphPad). Multiple comparisons were performed using the Kruskal–Wallis test and post-hoc Dunn’s test. Two-group comparison in Figure 4B was performed using the Mann–Whitney test.

## Data Availability

• Bulk RNA (GSE188822) and single-cell RNA-seq data (GSE188823) have been deposited in the NCBI Gene Expression Omnibus. • This paper does not report original code. • Additional Supplemental Items are available from Mendeley Data: http://dx.doi.org/10.17632/6pgb6rx8t8.1. • Any additional information required to reanalyze the data reported in this paper is available from the lead contact upon request.

## Supplemental information

Document S1. Figures S1–S3 and Tables S1–S4

Document S2. Article plus supplemental information
