# Supplementary material for: iPSC-derived mesenchymal cells that support alveolar organoid development
Source: Cell Rep Methods. 2022 Sep 19;2(10):100314. doi: 10.1016/j.crmeth.2022.100314 (PMC9606132; doi:10.1016/j.crmeth.2022.100314)
Supplement: Document S1. Figures S1–S3 and Tables S1–S4 [file mmc1.pdf]

Cell Reports Methods, Volume *2*

Supplemental information

iPSC-derived mesenchymal cells that support alveolar organoid development

Koji Tamai, Kouji Sakai, Haruka Yamaki, Keita Moriguchi, Koichi Igura, Shotaro Maehana, Takahiro Suezawa, Kazuaki Takehara, Masatoshi Hagiwara, Toyohiro Hirai, and Shimpei Gotoh

SUPPLEMENTAL INFORMATION

SUPPLEMENTAL FIGURES AND LEGENDS

Figure S1. Lineage marker expression of iMES and iMES-AOs. Related to Figure 1.

(A) RT-qPCR showing time-course changes in mesenchymal markers during iMES differentiation. iMES were differentiated from the 201B7 iPSC line. The relative gene expression was compared to 201B7 on day 0. Data are presented as mean ± SEM. (n=3 independent experiments). * *P*<0.05, Dunn's post-hoc test. (B) A phase-contrast image of AOs containing SFTPC-GFP$^+$ cells on day 14 from the initiation of 3D culture. iMES-AOs were generated by co-culture of lung progenitors derived from an SFTPC-GFP reporter iPSC line (B2-3) and iMES differentiated from the 201B7 iPSC line. Scale bar, 500 µm. (C) Immunofluorescence staining of iMES-AOs (P0). iMES-AOs were generated by co-culture of lung progenitors derived from an SFTPC-GFP reporter iPSC line (B2-3) and iMES differentiated from the 201B7 iPSC line. Scale bars, 50 µm. (D) RT-qPCR of AT2 and AT1 markers in HFLF-AOs, iMES-AOs, and HDF-AOs (P0). AOs were generated by the co-culture of lung progenitors derived from the SFTPC-GFP reporter (B2-3) iPSC line and HFLF, iMES differentiated from 201B7 and 604A1 iPSC lines, or HDF. Exogenous control RNA of human fetal lung at 17, 18, and 22 weeks of gestation was used (Agilent Technologies; #540177, lot 0006055802) to calculate relative AO gene expression. Data are presented as mean ± SEM. (n=3 independent experiments). * *P*<0.05 (Dunn's post hoc test).

Figure S2. Validation of generated iPSCs and the study of Wnt ligands and antagonists of TGFβ family ligands for SFTPC induction by the feeder-free method. Related to Figure 2.

(A) G-banding analysis of the karyotypes of HFA and GC23 iPSCs. (B) Flow cytometry showing the trilineage-differentiation potency of HFA and GC23 iPSCs. (C) Schematic diagram of an assay for fibroblast-free AOs induction. (D and E) Flow cytometry of the induction efficiency of SFTPC-GFP$^+$/EPCAM$^+$ cells and its quantification. Data are presented as mean ± SEM. * *P*<0.05, ** *P*<0.01 (Dunn's post hoc test). (2i: CHIR99021 and SB431542.) (F and G) The ratio of SFTPC-GFP$^+$/EPCAM$^+$ cells in each condition measured using flow cytometry. (3F: FST, FSTL1, and FSTL3. 4F: FST, FSTL1, FSTL3, and DCN)

Figure S3. Passage culture of SFTPC-GFP$^+$/EPCAM$^+$ cells in iMES-AOs and comparison of iMES-AOs and HFLF-AOs by the analysis of scRNA-seq data. Related to Figure 3.

(A) RT-qPCR of AT1 and AT2 markers in iMES-AOs (P0–P3). Exogenous control RNA of human fetal lung at 17, 18, and 22 weeks of gestation was used (Agilent Technologies; #540177, lot 0006055802) to calculate relative AO gene expression. Data are presented as mean ± SEM. (n=3, independent experiments.) * *P*<0.05 (Dunn's post hoc test). (B) UMAP plots of re-clustered epithelial cells except for mitotic cells and the representative lineage markers of each cluster. (C) Violin plots of representative genes expressed on mesenchymal cells except for mitotic cells.

Figure S1. Lineage marker expression of iMES and iMES-AOs. Related to Figure 1.

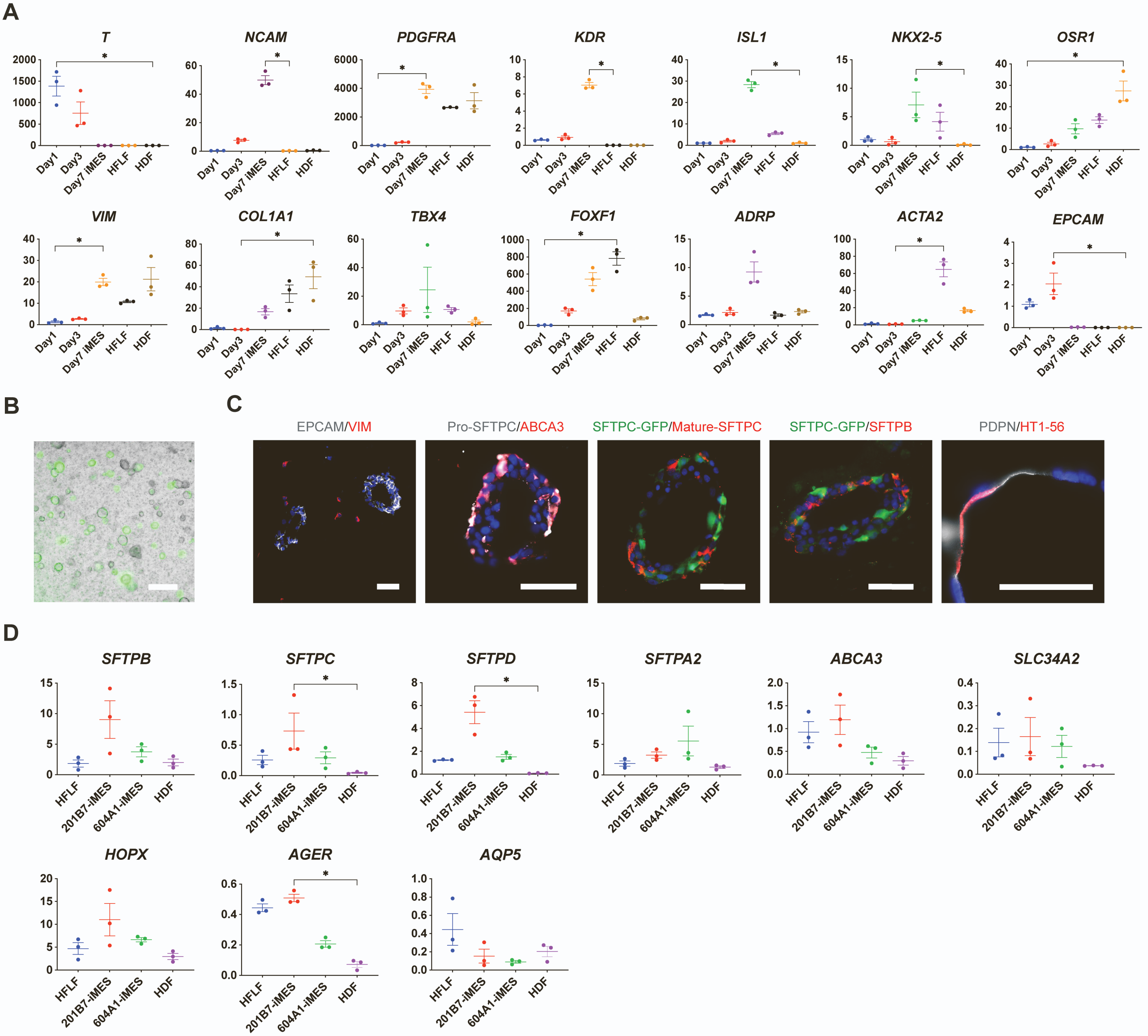

Figure S2. Validation of generated iPSCs and the study of Wnt ligands and antagonists of TGFβ family ligands for SFTPC induction by the feeder-free method. Related to Figure 2.

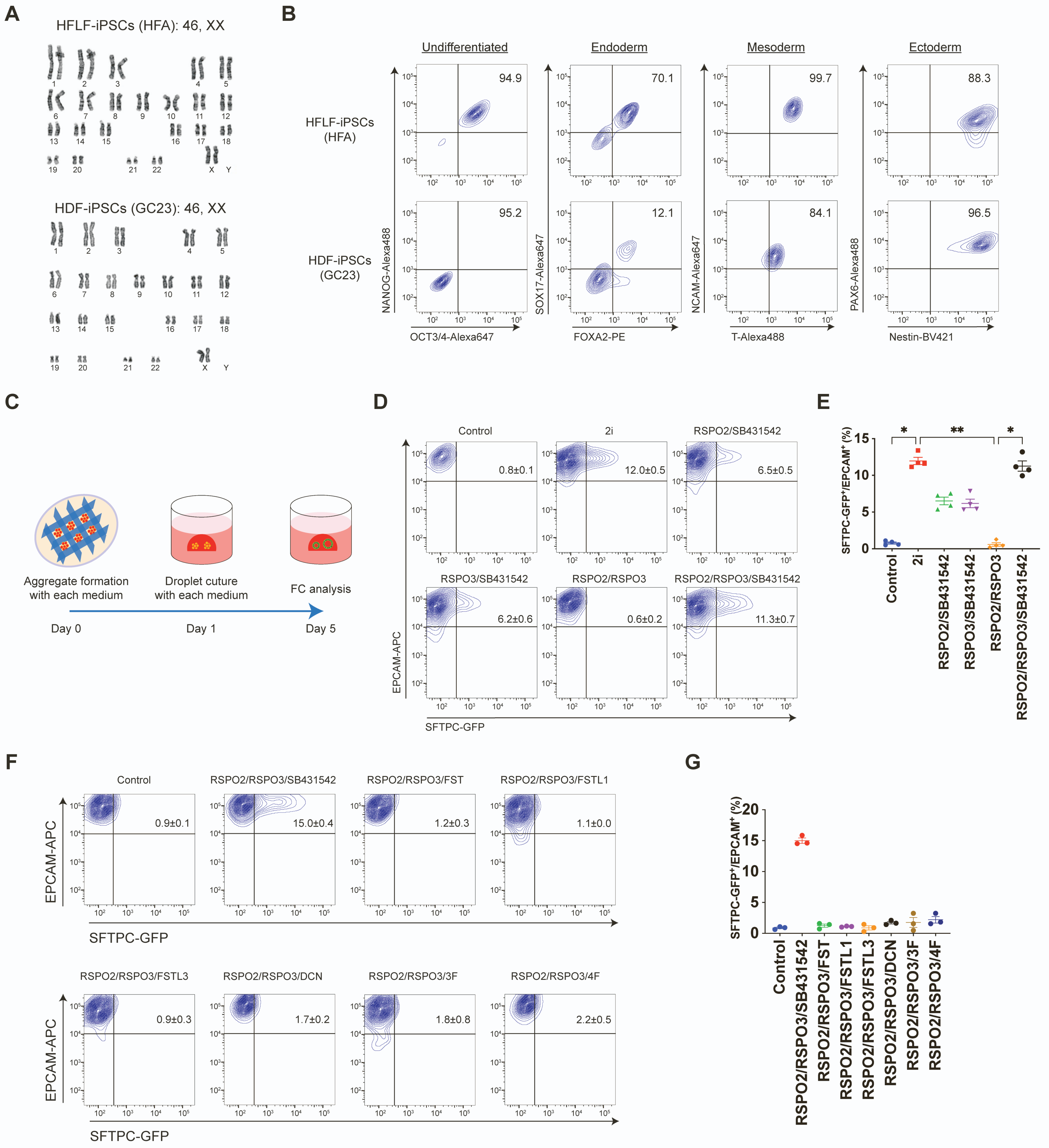

Figure S3. Passage culture of SFTPC-GFP⁺/EPCAM⁺ cells in iMES-AOs and comparison of iMES-AOs and HFLF-AOs by the analysis of scRNA-seq data. Related to Figure3.

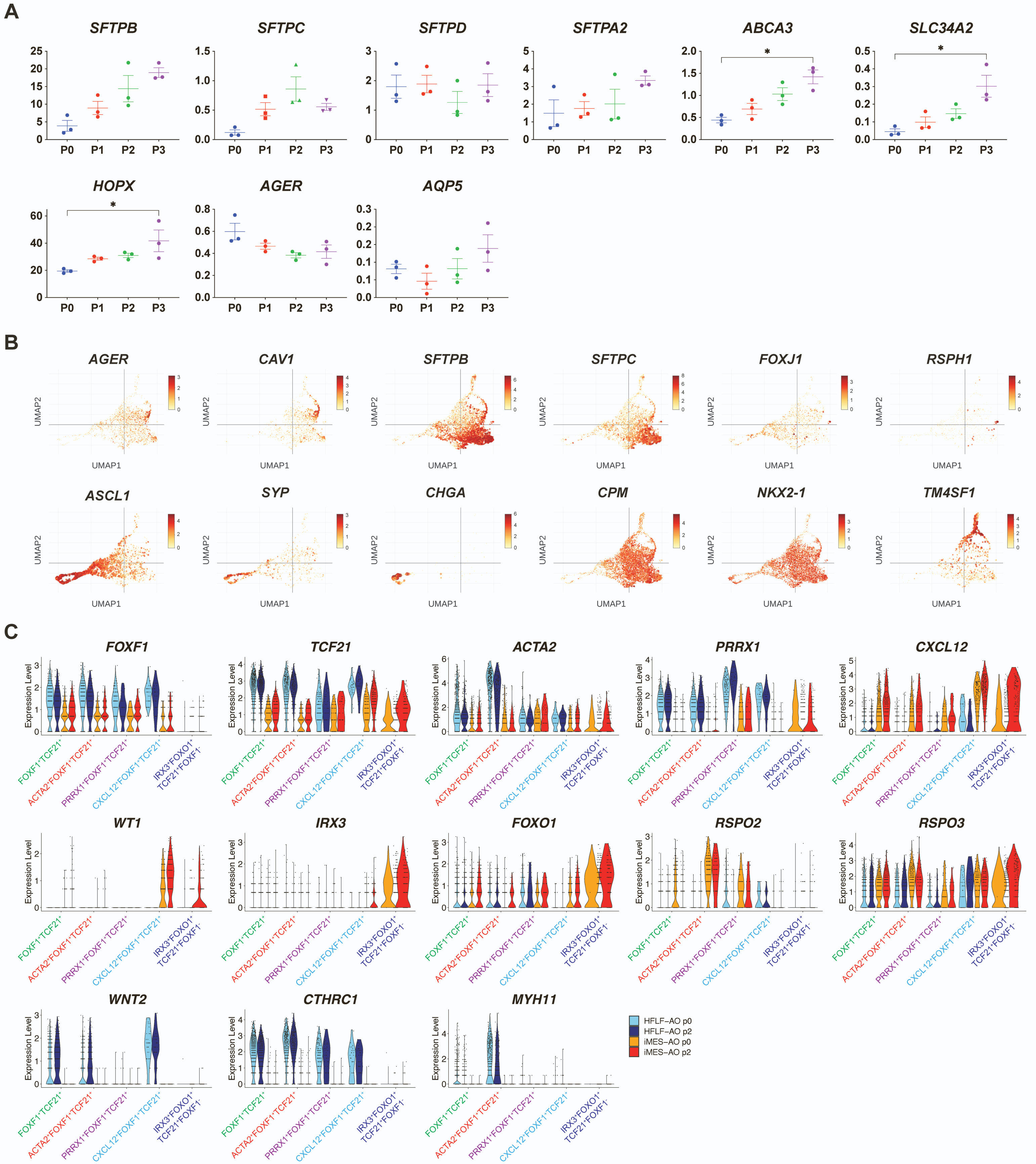

SUPPLEMENTAL TABLES WITH TITLES

Table S1. Content of each medium for differentiation of iPSCs into iMES. Related to STAR Methods.

	**Step1**	**Step2**
**Basal Medium**	StemPro 34<br>Gultamax (1x)<br>Penicillin/streptomycin (50 U/mL)	StemPro 34<br>Gultamax (1x)<br>Penicillin/streptomycin (50 U/mL)
**Chemicals/Cytokines**	ActivinA (15 ng/mL)<br>BMP4 (50 ng/mL)<br>CHIR99021 (1.5 µM)	ActivinA (3 ng/mL)<br>KGF (10 ng/mL)<br>BMP4 (25 ng/mL)<br>FGF2 (10 ng/mL)<br>FGF10 (10 ng/mL)

Table S2. Content of each medium for differentiation of iPSCs into LP. Related to STAR Methods.

	Endodermization medium (Step1)	Anteriorization medium (Step2)	Ventralization medium (Step3)	CFKD preconditioning medium (Step4)
Basal Medium	RPMI<br>B27 supplement (2%)<br>Penicillin/streptomycin (50 U/mL)	DMEM/F12<br>Glutamax<br>B27 supplement (2%)<br>L-ascorbic acid (0.05 mg/mL)<br>Monothioglycerol (0.4 mM)<br>Penicillin/streptomycin (50 U/mL)		
Chemicals /Cytokines	Activin A (100 ng/mL)<br>CHIR99021 (1.0 µM)<br>Y-27632 (Day0; 10 µM)<br>*Sodium butyrate (NaB)* (Day 1; 0.25 mM, Days 2 and 4; 0.125 mM)	Noggin (100 ng/mL)<br>SB431542 (10 µM)	ATRA (depends on cell lines)<br>B2-3: 0.05-0.5 µM<br>CHIR99021 (3 µM)<br>BMP4 (20 ng/mL)	CHIR99021 (3 µM)<br>FGF10 (10 ng/mL)<br>KGF (10 ng/mL)<br>DAPT (20 µM)

	Alveolarization medium
Basal Medium	Ham's F12<br>B27 supplement (1%)<br>BSA (0.25%)<br>HEPES (15 mM)<br>$CaCl_2$ (0.8 mM)<br>ITS premix (0.1%)<br>Penicillin /streptomycin (50 U/mL)
Chemicals /Cytokines	Dexamethasone (50 nM)<br>IBMX (100 µM)<br>KGF (10 ng/mL)<br>8-Br-cAMP (100 µM)<br>Y-27632 (Day 0; 10 µM, every day for FF-AOs.)

Table S3. Candidates of Wnt ligands and antagonists of TGF-β family ligands. Related to Figure 2.

Gene candidates

Wnt ligands			Antagonists of TGFβ family ligands
RSPO1	WNT5A	WNT10A	FST
RSPO2	WNT5B	WNT10B	FSTL1
RSPO3	WNT6	WNT11	FSTL3
WNT1	WNT7A	WNT16	DCN
WNT2	WNT7B		WFIKKN1
WNT2B	WNT8A		WFIKKN2
WNT3	WNT8B		A2M
WNT3A	WNT9A		EMILIN1
WNT4	WNT9B		

Gene candidates showing a maximum average TPM of 3 replicates >20 in at least one group among four mesenchymal cell lines in post-3D culture condition

Gene symbol	Post 3D culture HFLF-iMES	Post 3D culture HDF-iMES	Post 3D culture HFLF	Post 3D culture HDF
A2M	1.6	2.5	2440.8	14.3
DCN	5772.4	4090.6	2557.2	16111.3
EMILIN1	76.9	36.2	509.4	182.2
FST	15.3	35.5	116.5	84.5
FSTL1	364.3	331.9	1412.4	1603.1
FSTL3	55.9	55.8	174.9	87.7
RSPO2	176.4	141.7	14.1	0.3
RSPO3	59.7	37.5	39.3	0.7
WNT11	25.9	58.9	19.2	0.8
WNT2	0.3	0.6	67.9	0.9
WNT5A	9.4	17.3	43.8	559.2
WNT5B	16.3	22.9	12.3	30.2

Table S4. Primers for RT-qPCR used in the present study. Related to STAR Methods.

Gene	Forward	Reverse
ACTB	CAATGTGGCCGAGGACTTTG	CATTCTCCTTAGAGAGAAGTGG
T	TGAGCCTCGAATCCACATAGTG	AAGAGCTGTGATCTCCTCGTTC
NCAM	CAACCTGTGTGGAAAAGCCG	CATGGTTTGGGGTCCTCTCC
PDGFRA	TGGGCACGCTCTTTACTCC	ATTAGGCTCAGCCCTGTGAG
KDR	GGCGGCACGAAATATCCTCT	GGAGGCGAGCATCTCCTTTT
NKX2-5	AAAAGAAAGAGCTGTGCGCG	CTCCAGCTCATAGACCTGCG
ISL1	GCAGCAGCCCAATGACAAAA	GTTAGCCTGTAAGCCACCGT
OSR1	CGAAATGGGCAGCAAAAC	ATACAGGTTGGGCAGATGGT
VIM	CGGGAGAAATTGCAGGAGGA	AAGGTCAAGACGTGCCAGAG
COL1A1	CCCCGAGGCTCTGAAGGTC	GGAGCACCATTGGCACCTTT
FOXF1	AGCCGTATCTGCACCAGAAC	TCCTTTCGGTCACACATGCT
TBX4	CACCCTCGCCACATCTAAA	CGTCAGTCCAGTTCTCCACA
ADRP	TCAGCTCCATTCTACTGTTCACC	CCTGAATTTTCTGATTGGCAC
ACTA2	GGGTGACGAAGCACAGAGCA	CTTCAGGGGCAACACGAAGC
EPCAM	AGAACCTACTGGATCATCATTGAACTAA	CGCGTTGTGATCTCCTTCTG
SFTPB	GAGCCGATGACCTATGCCAAG	AGCAGCTTCAAGGGGAGGA
SFTPC	GCAAAGAGGTCCTGATGGAG	TGTTTCTGGCTCATGTGGAG
SFTPD	AGGAGCAAAGGGAGAAAGTGGG	CAGCTGTGCCTCCGTAAATGG
SFTPA2	AAGCAGCTGGAGGCTCTGT	CCATCAAGATGAGGGTGAGG
ABCA3	TCTCCTTCAGCTTCATGGTCAG	TGGCTCAGAGTCATCCAGTTG
SLC34A2	TCGCCACTGTCATCAAGAAG	CTCTGTACGATGAAGGTCATGC
AGER	GCCACTGGTGCTGAAGTGTA	TGGTCTCCTTTCCATTCCTG
HOPX	TCAACAAGGTCGACAAGCAC	TCTGTGACGGATCTGCACTC
AQP5	CTGTCCATTGGCCTGTCTGTC	GGCTCATACGTGCCTTTGATG
